# Evolution Mechanism and High-Precision Quantitative Identification of MFL Signals from Defects Under Supersaturated Magnetization Conditions

**DOI:** 10.3390/s26103092

**Published:** 2026-05-13

**Authors:** Huiqi Zou, Jiuxin Wang, Qi Dong, Dingze Lu, Yurong Du, Yaoheng Su

**Affiliations:** 1 School of Science, Xi’an Polytechnic University, Xi’an 710048, Chinaludingze@whu.edu.cn (D.L.);; 2 Shaanxi Special Equipment Inspection and Testing Institute, Xi’an 710048, China; 220811005@stu.xpu.edu.cn; 3 School of Optoelectronic Science and Intelligent Instrumentation, Xi’an University of Technology, Xi’an 710048, China

**Keywords:** magnetic flux leakage testing, supersaturated magnetization, finite element simulation, signal characterization model, signal correction method

## Abstract

Magnetic flux leakage (MFL) testing is a critical non-destructive testing (NDT) method for ensuring the safety of ferromagnetic storage and transportation equipment. However, existing research has predominantly focused on weak or saturated magnetization states, leaving the characteristic laws and physical mechanisms of defect signals under supersaturated magnetization conditions unclear. To address this gap, this paper systematically investigates the MFL signal evolution mechanism and develops a high-precision quantitative identification method for defects under supersaturated magnetization conditions through finite element simulation, theoretical modeling, and experimental validation. First, a three-dimensional (3D) finite element model for MFL testing is established using COMSOL Multiphysics. The regulatory effects of key parameters—sensor lift-off value, defect burial depth, length, and depth—on the peak values and distribution characteristics of axial and radial MFL signals are revealed, a signal peak characterization model for each parameter and their adjusted $R^{2}$ is obtained via fitting, and the detection capability of the detector for defects with different shapes is simultaneously verified. Furthermore, actual detection is conducted on three crack defects of different sizes, and the analysis results indicate that the characterization models of each parameter obtained from the simulation exhibit high accuracy. The results show that MFL signal intensity under supersaturated magnetization conditions is significantly enhanced compared to that under saturated magnetization conditions. Furthermore, to improve defect length measurement accuracy, a signal correction method based on the midpoint of extreme values of the second derivative of axial signals is proposed. By compensating for peak offsets caused by factors like magnetic field diffusion, this method reduces the maximum defect length identification error from 14.25% (pre-correction) to below 0.3%. This study elucidates the coupling influence mechanism of multi-physical parameters on MFL signals under supersaturated magnetization conditions. The proposed high-precision signal correction method provides a novel theoretical basis and technical approach for the accurate quantification and inversion of defects in complex operating conditions.

## 1. Introduction

At present, petroleum and natural gas have become cornerstone energy resources underpinning the economic development of modern society [1,2]. This dependency has directly driven the widespread deployment of petrochemical storage and transportation infrastructure, including storage tanks and oil–gas pipelines, which are used for hydrocarbon storage and distribution [3,4]. However, during long-term service, this equipment is susceptible to defects such as cracks and holes, which may result from mechanical damage, corrosion, or other detrimental factors [5]. Timely detection of these defects is critical; failure to do so can lead to oil and gas leaks, resulting in safety incidents such as combustion and explosions, which can cause severe casualties and significant economic losses [6]. To mitigate these safety risks, regular and effective inspections of in-service equipment are essential for safeguarding national security and ensuring stable economic development [1]. Non-destructive testing (NDT) encompasses various physical and chemical methods used to inspect and evaluate the continuity, integrity, reliability, and safety of workpieces without compromising their operational performance or internal structures. Conducting regular inspections and evaluations of in-service petrochemical storage and transportation equipment through NDT methods is an effective strategy to ensure their safety and reliability [7,8]. Among the various NDT techniques, magnetic flux leakage (MFL) testing exhibits high effectiveness for ferromagnetic materials and has been widely adopted in industrial inspections due to its ease of operation, high detection efficiency, and excellent environmental adaptability.

Conventional NDT methods mainly include radiographic testing (RT), ultrasonic testing (UT), magnetic particle testing (MT), penetrant testing (PT), eddy current testing (ECT), and magnetic flux leakage (MFL) testing [9]. RT employs high-penetration high-frequency electromagnetic waves, which induce ionization effects, fluorescence, and photochemical reactions in the inspected objects. The location and size of defects are determined by detecting the intensity variation of transmitted radiation caused by these interactions [10]. Although this method can provide intuitive images of defects, it poses potential radiation hazards to humans and the environment. In UT, defect location and size are identified by analyzing the received reflected wave signals. UT features high penetration depth, enabling the detection of deep-seated defects; however, it exhibits low sensitivity to surface defects and requires the use of coupling agents during testing, which complicates the inspection process [6]. MT detects defects by leveraging the magnetic flux leakage characteristics of ferromagnetic materials. When a ferromagnetic specimen is magnetized, the magnetic field lines are distorted due to the difference in magnetic permeability between the defect and the specimen, forming a leakage magnetic field. When a layer of magnetic particles is applied to the material’s surface, these particles will accumulate spontaneously in the leakage field area, facilitating defect identification. Nonetheless, MT can only locate defects and does not quantitatively characterize parameters such as defect depth and size; furthermore, materials like magnetic particles or magnetic suspensions are prone to causing environmental pollution. In PT, fluorescent or colored dyes (penetrants and developers) are sequentially applied to the specimen surface. The penetrants infiltrate the interiors of defects through capillary action, and under black or white light illumination the residues of the penetrant within the defects reveal yellow-green or red traces, which facilitate the detection of defect morphology and distribution [11]. However, PT is only effective for surface-breaking defects, and the penetrants and developers employed may contribute to environmental pollution. In ECT, an alternating magnetic field generated by an excitation coil induces eddy currents in the specimen that then produce secondary magnetic fields. Defect detection is achieved by analyzing the signals from these secondary fields received by a detection coil. Due to the skin effect, ECT is limited to detecting surface and near-surface defects in the specimen. MFL identifies defects by capturing the leakage magnetic fields that they create. When a ferromagnetic specimen is magnetized to saturation, the magnetic resistance at the defect is higher than that of the specimen itself, resulting in distortion of the magnetic field lines that bypass the defect and leakage into the surrounding air, thereby generating a leakage magnetic field. Defect characteristics can be identified by collecting and analyzing magnetic field signals using magnetic sensors. Petrochemical storage and transportation equipment is typically constructed from ferromagnetic metal materials. Compared to other NDT technologies, MFL presents distinct advantages, including environmental friendliness, no need for coupling agents, operational simplicity and convenience, high reliability, and applicability to both surface and internal defects [12], thereby demonstrating greater application advantages.

Considerable advances have been made in theoretical and technical research regarding MFL testing. Frankowski et al. [13] compared the DC magnetic method for detecting reinforced concrete under non-magnetization, opposite-pole magnetization, and same-pole magnetization conditions. It was found that opposite-pole magnetization can achieve higher signal amplitude and signal-to-noise ratio (SNR) and can reliably identify complex steel bar arrangements, while the signals under opposite-pole magnetization and non-magnetization conditions are weak with blurred characteristics. By means of a nonlinear magnetic equivalent circuit model, Kara [14] found that near-saturation magnetization is a prerequisite for effective MFL detection. Under this condition, a high magnetic flux density is exhibited in the pole region. Excessive excitation of leakage flux in the slot results in the distortion of defect signals. Shi [15] established a quantitative propagation model for magnetic flux leakage (MFL) signals based on the Non-Uniform Magnetic Charge Model (NUMCM). By introducing a demagnetization factor, this model quantitatively characterized the attenuation behavior of outer-wall defect signals under near-saturated magnetization conditions, providing a theoretical basis for quantitative defect detection under near-saturated magnetization conditions. It was found by Liu [16], via finite element simulation and experimental research, that the specimen enters an supersaturated magnetization state at small pole pitch. A new double-peak-valley (DPV) phenomenon is observed in the radial magnetic flux leakage signals. This phenomenon arises from the interaction between magnetic diffusion and compression. In magnetic flux leakage testing, the magnetic field distribution and detection capability are directly affected by the pole configuration of the magnetizer. In magnetic flux leakage (MFL) testing, magnetic field distribution and detection capability are both directly influenced by the pole configuration of the magnetizer. Two configurations, namely same-pole and opposite-pole arrangements, were systematically compared by Ahmad et al. [17]. The opposite-pole configuration is adopted in conventional MFL testing, where magnetic flux is confined within the tested material, and leakage magnetic flux is generated when defects are present. In contrast, a forced MFL effect is produced by the newly proposed same-pole configuration, which can effectively detect magnetic inclusions in non-magnetic matrices. A lightweight four-pole magnetizer incorporating both same-pole and opposite-pole structures was further developed by Pham et al. [18]. The interval angle θ of same poles acts as a key parameter governing magnetic field uniformity. The optimal angle θ is determined to be 30–40°. Under this condition, both the amplitude and the uniformity of the magnetic field are verified to be superior to those of the traditional opposed two-pole configuration. Gao et al. [19] established a defect leakage magnetic field based on the uneven magnetic charge distribution of magnetic dipoles, elucidating the influence of various defect sizes on MFL signals. Zhang [20] proposed an algorithm for assessing the depth field of surface defects in single-layer steel pipes by constructing a discrete magnetic dipole model, thereby enhancing the accuracy of defect reconstruction in MFL testing. Yang et al. [21] investigated variation in the signal-to-noise ratio (SNR) of MFL signals with different ferromagnetic stripping media, with the objective of alleviating the high noise interference caused by the substantial surface roughness of workpieces. The limitations of MFL technology in detecting small defects were explored by Coleman et al. [22]. The effects of material properties, defect morphology, signal-to-noise ratio, and operating parameters on sensitivity improvement were analyzed. Defects well below the nominal resolution threshold can be identified under certain conditions. Lam [23] proposed a systematic design method for the yoke magnetization element of an MFL testing system, which maximizes magnetic flux leakage and reduces the weight and volume of the yoke, thus ensuring sufficient magnetization capability. Liu et al. [24] derived the analytical relationship between lift-off value and noise and developed an image-based denoising method by summarizing the signal and morphological characteristics of low-frequency signals, crosstalk noise, and jitter noise. This method effectively improves the SNR of MFL signals, enabling more reliable detection of low-frequency signals. A handheld MFL scanner based on a quantum well Hall effect sensor array was developed by Biruu et al. [25] The defect detection performance under DC and AC magnetization conditions was investigated separately. Surface defects, subsurface defects, and microstructural variations can be effectively identified. Hosseingholizadeh et al. [26] constructed an alternating current (AC) MFL testing system by winding a coil on an iron core and applying AC power, which enhances the accuracy of defect type identification. Gong et al. [27] designed a circular sensor array based on MFL technology to quantify the angle, width, and length of cracks. Ru et al. [28] proposed an electromagnetic structural coupling sensing system that integrates alternating magnetic field and MFL testing, making it suitable for detecting defects in various directions, as well as surface and subsurface defects in metal materials. Chang [29] proposed a magnetoelectric-ultrasonic hybrid transducer that combines electromagnetic and ultrasonic testing mechanisms. By exciting the transducer with double-pulse signals of different pulse widths, this transducer achieves simultaneous detection of surface and internal defects in metals. A method for defect size estimation from magnetic flux leakage signals based on a convolutional neural network (CNN) was proposed by Wang et al. [30]. The model is composed of two modules: defect classification and defect size regression. Data fusion and feature extraction of three-axis signals are implemented by the former module. Size estimation of different defects is realized by the latter module through seven CNNs. MFL defect sizes in pipelines can thus be effectively identified. Shen et al. [31] introduced a task-oriented physical collaboration network aimed at quantifying the size of defects corresponding to MFL signals, achieving a detection accuracy of 96%.

Currently, MFL testing technology is primarily advancing in three directions: first, enhancing defect detection accuracy through MFL signal amplification, denoising, and other techniques; second, addressing the limitations of single detection methods by integrating MFL with other technologies; and third, achieving defect size inversion through algorithms and related methodologies.

Despite the gradual maturation of current magnetic flux leakage (MFL) testing technology, most studies have primarily concentrated on weak or saturated magnetization, with limited investigations conducted under supersaturated magnetization conditions. Furthermore, the existing literature lacks clarity regarding the correlation between MFL signals and various characteristic parameters of defects, as well as the underlying physical mechanisms. Additionally, the detection accuracy for defect dimensions requires further enhancement. To address these limitations, this study investigates the correlation between magnetic field signals and various characteristic parameters of defects under supersaturated magnetization, elucidating the corresponding physical mechanisms. The developed peak characterization models of detection signals for each characteristic parameter establish a reliable foundation for defect inversion. Furthermore, a signal correction method based on the midpoint of extreme values of the second derivative of axial signals is proposed, effectively maintaining the defect length identification error within 0.3%.

This work systematically elaborates on the fundamental principles of MFL testing and its simulation methods from a theoretical perspective. Building on this foundation, this study compares the amplitude characteristics of MFL signals under various magnetization states to determine the optimal magnetization conditions for testing. This analysis thoroughly reveals the correlation patterns between and physical essence of the testing magnetic field signals and various characteristic parameters, and analyzes the influence of specimen size on the detection signals, and tests the recognition capability of the detector for defects of different shapes. Furthermore, a three-dimensional magnetic dipole model is introduced to validate the simulation results, and a signal correction method based on the midpoint of extreme values in the second derivative of axial signals is proposed. Subsequently, experiments are conducted to verify the accuracy of both the simulation data and the constructed characterization model of testing signals. This study not only deepens understanding of the evolution mechanism of magnetic flux leakage signals, but also provides theoretical support and technical pathways for high-precision and high-reliability defect quantitative identification.

## 2. Principles and Theoretical Analysis of Simulation

### 2.1. Analysis of MFL Testing Principles

For MFL testing technology, the specimen first needs to be magnetized to saturation by an MFL detector. A closed magnetic circuit is formed by the magnetic source, specimen, and yoke inside the detector. Subsequently, magnetic field signals are collected by the magnetic sensors on the detector, the presence of defects is determined, and their characteristics are identified by analyzing the features of MFL signals [16]. Based on the magnetic charge model, collections of magnetic charges exist on the two magnetic pole end faces of the permanent magnet. It is assumed that $n_{1}$ magnetic charges are accumulated on the positive and negative end faces of the permanent magnet, respectively. The magnetic flux density $B_{p}$ excited by the permanent magnetic field at a certain point in space is expressed as

(1) $$B_{p}=\frac{\mu_{0}}{4\pi }\sum_{i=1}^{n_{1}}\frac{q_{p_{i}}r_{i}}{{\left|r_{i}\right|}^{3}}$$

where $\mu_{0}$ is the vacuum permeability, with a value of $4\pi \times 10^{7}$; $q_{pi}$ is the magnetic charge quantity of the i-th magnetic charge on the end face of the permanent magnet, whose magnitude is related to the magnetization intensity of the magnet; and $r_{i}$ is the position vector from the i-th magnetic charge on the end face of the magnet to the spatial detection point.

When a defect exists within the magnetized region of the specimen, its higher magnetic resistance causes the magnetic field lines to distort and bypass it. If this distortion is sufficiently severe, some of the magnetic field lines will leak into the surrounding air, thereby generating a leakage magnetic field. According to the equivalent magnetic charge model, this phenomenon can be characterized by equal amounts of opposite-polarity magnetic charges accumulating on the left and right wall surfaces of the defect. It is assumed that the accumulated magnetic charge quantity is $n_{2}$, and the magnetic flux density $B_{l}$ of the leakage magnetic field generated by the defect at a certain point in space is expressed as

(2) $$B_{l}=\frac{\mu_{0}}{4\pi }\sum_{k=1}^{n_{2}}\frac{q_{lk}r_{k}}{{\left|r_{k}\right|}^{3}}$$

**Figure 1:**
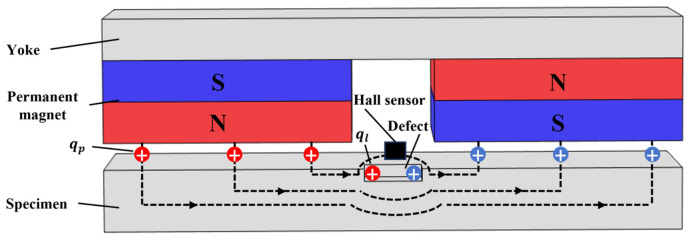
Schematic diagram of MFL testing.

The magnetic sensor is placed between the two magnets. It measures the total magnetic flux density B. In an idealized model, if the detected flux density is $B=B_{p}$, the inspected region is considered defect-free. Conversely, if the measurement yields $B=B_{p}+B_{l}$, the presence of a defect is confirmed. Therefore, the presence or absence of defects can be determined by checking whether a leakage magnetic field exists in the signals collected by the sensor, and defect characteristics can be identified based on signal features. A schematic diagram of MFL testing is shown in Figure 1.

### 2.2. Theoretical Analysis of Simulation

To investigate the influence of defect characteristics on MFL signals, a finite element model for MFL testing is established in this study, based on the mfnc (magnetic field, no currents) physics interface in COMSOL Multiphysics 6.3. This interface is designed for magnetostatic problems without conduction currents, involving only a magnetization vector field. Its governing equations are derived from magnetostatic simplification of Maxwell’s equations.

Under conditions of a static magnetic field and no current, Maxwell–Ampère’s law can be simplified into a curl form, which indicates that the magnetic field strength H is an irrotational field ($\nabla \times \left(\frac{1}{\mu_{0}}B-M\right)=\nabla \times H=0$). Meanwhile, a magnetic scalar potential $V_{m}$ is introduced for its scalar description ($H=-\nabla V_{m}$). Furthermore, based on the basic constitutive relation of magnetic field strength ($B=\mu_{0}(H+M)$), and combined with the divergence constraint of static magnetic field in Gauss’s magnetic law (∇•B=0), the static magnetic field equation under mfnc can be derived, which is expressed as

(3) $$\left\{\begin{array}{c} \begin{array}{c} \nabla \times \left(\frac{1}{\mu_{0}}B-M\right)=\nabla \times H=0\\ H=-\nabla V_{m} \end{array}\\ \begin{array}{c} B=\mu_{0}\left(H+M\right)\\ \nabla •B=0 \end{array} \end{array}\right.\to -\nabla \cdot \left(\mu_{0}\left(\nabla V_{m}-M\right)\right)=0$$

where ∇ is the vector differential operator, B is the magnetic flux density, M is the magnetization vector field, H is the magnetic field strength, $\mu_{0}$ is the vacuum permeability, and $V_{m}$ is the magnetic scalar potential.

## 3. Analysis of Finite Element Simulation Results

**Figure 2:**
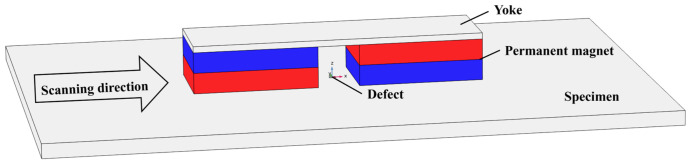
MFL testing model.

The MFL testing model constructed in the simulation in the COMSOL mfnc physical field is shown in Figure 2. The outer boundary of the entire solution domain is set as the magnetic insulation boundary condition, which is expressed as n•B=0. The model is meshed with tetrahedral elements, the maximum element size was determined to be 33 mm. The minimum element size was determined to be 2.4 mm. A total of 214,274 elements are included in the generated mesh. In the model, the magnetization unit in the detector consists of permanent magnets and yokes. The geometric dimensions (length × width × height) of the permanent magnet are 60 mm × 40 mm × 10 mm, with a residual magnetic flux density magnitude of 1.21 T; the distance between the two permanent magnets is set to 40 mm. The dimensions (length × width × height) of the yoke are 160 mm × 40 mm × 3 mm, with a relative permeability of 4000 and an electrical conductivity of 10.295 MS/m. The specimen is made of low-carbon steel 1010, with a thickness of 7 mm, a relative permeability of 300, and an electrical conductivity of 8.41 MS/m. The Hall sensor used for collecting magnetic field signals has a diameter of 16 mm. The standard geometric dimensions (length × width × height) of the designed defect are 8 mm × 2 mm × 2 mm, the standard burial depth is 0.2 mm, and the standard lift-off value of the Hall sensor is 0.1 mm. During the simulation, by changing only one parameter while keeping the other parameters at their standard values, the influence of changes in different characteristic parameters on MFL detection signals is investigated. Furthermore, the peak characterization models of detection signals corresponding to different parameters are obtained through fitting, which provides a reliable basis for the inversion of characteristic parameters.

### 3.1. Selection of the Pole Configuration

**Figure 3:**
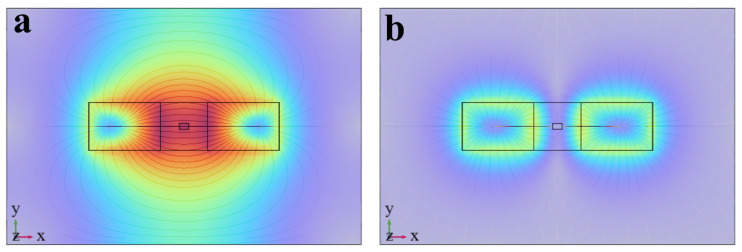
Magnetization effects under different pole configurations: (**a**) opposite-pole magnetization, (**b**) same-pole magnetization. (Colors from red to blue represent a decrease in magnetic flux density).

In magnetic flux leakage testing, the uniformity and strength of the internal magnetization field are directly determined by the pole configuration of the magnetizer. This is one of the key factors affecting the amplitude and SNR of defect MFL signals [32]. Based on different polar arrangements, magnetization methods in MFL testing are classified into two categories: same-pole magnetization and opposite-pole magnetization. The two magnetization schemes for the same magnet size are compared in this paper. High-efficiency magnetization states are investigated. The thermal magnetization results are shown in Figure 3.

The simulation results show that magnetization intensity in the specimen is significantly higher under opposite-pole magnetization conditions than under same-pole magnetization conditions. Under same-pole magnetization conditions, the same polarity is used for adjacent magnetic poles. Repulsion and divergence of magnetic flux lines occur in the specimen. Large magnetic flux leakage is caused. The magnetization intensity inside the specimen is low and non-uniform. Under opposite-pole magnetization conditions, a closed magnetic circuit is formed by magnetic attraction. Magnetic flux lines are concentrated more intensively through the specimen. Flux leakage loss in the air domain is effectively reduced. The overall magnetization effect and field uniformity are significantly improved [33].

In summary, the opposite-pole magnetization scheme exhibits obvious advantages in magnetization intensity, magnetic field uniformity, and magnetic energy utilization. Efficient magnetization of the specimen is realized.

### 3.2. Magnetization State

**Figure 4:**
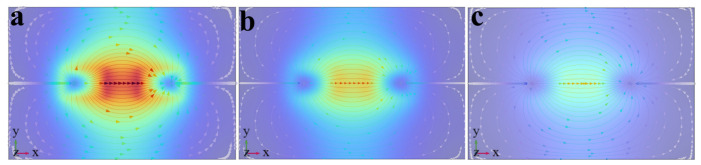
Heat map of magnetic flux density in different magnetization states: (**a**) supersaturated magnetization, (**b**) saturation magnetization, (**c**) near-saturation magnetization. (Colors from red to blue represent a decrease in magnetic flux density).

**Figure 5:**
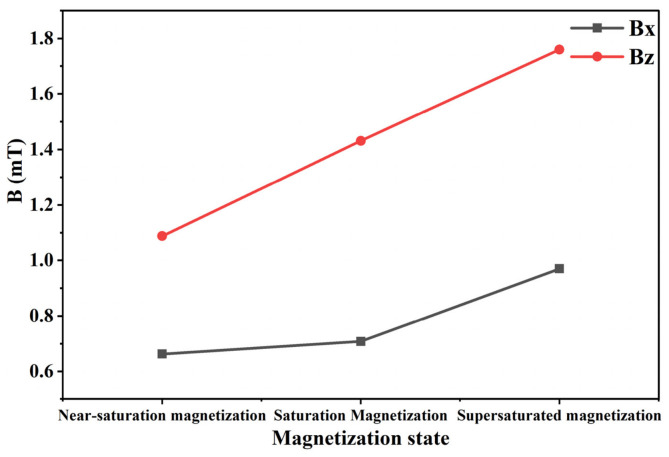
Axial and radial MFL signals in different magnetization states.

In MFL testing, magnetization intensity is a key factor affecting defect detection capability. During magnetization of the specimen, the internally randomly arranged magnetic domains are rearranged under the torque action of the external magnetic field and eventually achieve orderly alignment in the direction of the external magnetic field. The orientation degree of magnetic domains directly affects the generation mechanism and signal characteristics of the leakage magnetic field at defects [34,35]. At present, studies on MFL testing for steel materials are mainly conducted in three magnetization states: saturation magnetization, near-saturation magnetization, and supersaturated magnetization. Saturation magnetization is defined as magnetization energy 1.5–2 times the intrinsic coercivity of magnetic materials. Near-saturation magnetization refers to a magnetization level of about 80% of magnetic saturation. Over-saturation magnetization is regarded as an ultra-strong state where magnetization energy is more than three times the intrinsic coercivity. To explore the magnetization state that maximizes MFL signals, the magnetization state of the specimen is regulated in COMSOL by adjusting the distance between the two permanent magnets of the magnetizer. For low-carbon steel 1010, the intrinsic coercivity is 360–680 A/m. According to the corresponding relationship between coercivity and magnetization intensity in its magnetic characteristic curve (B-H curve), the corresponding saturation magnetization intensity ranges from 1.60 T to 2.10 T. Based on this, the specimen is magnetized to 1.55 T, 1.85 T, and 3.00 T to achieve three magnetization states: near-saturation magnetization, saturation magnetization, and supersaturated magnetization. The magnetic flux density heat maps in different states are shown in Figure 4. In addition, the total detection signal and permanent magnetic field signal in different magnetization states are extracted. The axial $B_{x}$ and radial $B_{z}$ components of the leakage magnetic field are obtained from their difference. The axial and radial leakage magnetic field signals in different magnetization states are shown in Figure 5.

**Figure 6:**
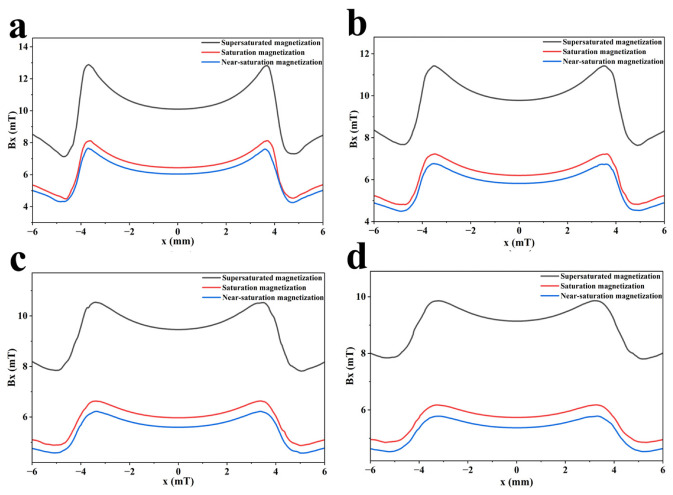
Axial signals in different magnetization states at the same lift-off value: (**a**) lift-off value = 0.1 mm, (**b**) lift-off value = 0.3 mm, (**c**) lift-off value = 0.5 mm, (**d**) lift-off value = 0.7 mm.

**Figure 7:**
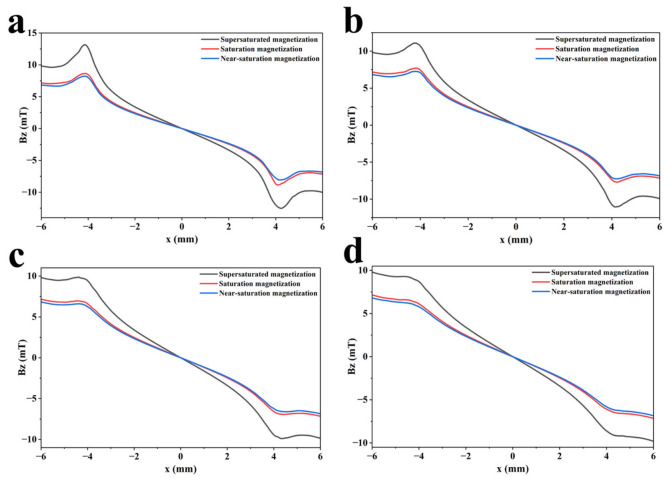
Radial signals in different magnetization states at the same lift-off value: (**a**) lift-off value = 0.1 mm, (**b**) lift-off value = 0.3 mm, (**c**) lift-off value = 0.5 mm, (**d**) lift-off value = 0.7 mm.

However, the background magnetic field increases as the magnetization state increases. There are thus adverse effects on the lift-off value [36]. To verify the effectiveness of over-saturation magnetization and study its effect on lift-off value, comparative tests are carried out at lift-off values of 0.1, 0.3, 0.5, and 0.7 mm. The signal characteristics of the three magnetization states are analyzed. The detection results are shown in Figure 6 and Figure 7.

As shown in Figure 4 and Figure 5, the MFL signal amplitude exhibits an increasing trend with rising magnetization intensity. Notably, the MFL signal amplitude under supersaturated magnetization conditions is significantly increased compared with that under saturated magnetization conditions. In addition, it can be concluded from Figure 6 and Figure 7 that, compared with near-saturated magnetization and saturated magnetization, the supersaturated magnetization state can produce stronger detection signals and more prominent defect signal characteristics at the same lift-off value, leading to a remarkable improvement in defect recognition capability. Therefore, to achieve optimal detection during MFL testing, MFL testing should be performed on the specimen in a supersaturated magnetization state.

### 3.3. Magnetic Field Distribution Heat Map

**Figure 8:**
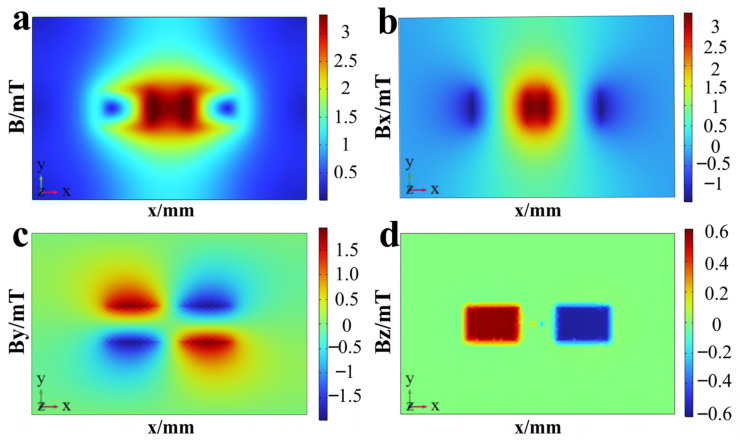
Magnetic field distribution heat maps in different directions: (**a**) total magnetic field signal B, (**b**) axial magnetic field signal $B_{x}$, (**c**) circumferential magnetic field signal $B_{y}$, (**d**) radial magnetic field signal $B_{z}$.

**Figure 9:**
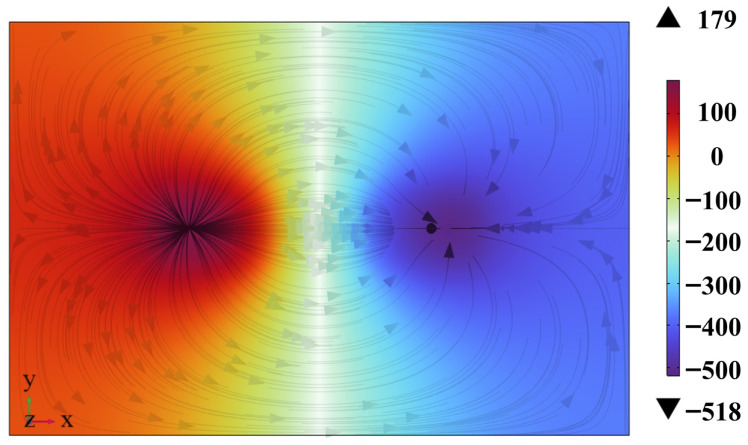
Magnetic scalar potential map of plane xoy.

In MFL testing research, the heat map of magnetic field signal distribution can intuitively characterize the spatial distribution characteristics of the specimen’s multi-directional magnetic field. With each parameter set to the standard value, the distribution heat maps of magnetic fields in different directions within the cross-section (plane xoy) of the magnetic source are shown in Figure 8. The magnetic scalar potential maps of the xoy plane obtained simultaneously are shown in Figure 9. Streamlines represent the magnetic field in A/m. The direction of magnetic flux lines is indicated by streamline arrows. The magnetic field strength is reflected by streamline density. The distribution and direction of magnetic field signals are clearly shown in the maps. An intuitive basis is provided for the analysis of $B_{x}$, $B_{y}$, and $B_{z}$ signals. The magnetic scalar potential map can intuitively present the high and low signal distribution of the magnetic field, as well as the direction of the magnetic field signal. To clearly display the characteristics of the magnetic field signal, the magnetic scalar potential map of plane xoy obtained synchronously is shown in Figure 5. It can be seen from the figures that the total magnetic field B exhibits a symmetrical distribution with the central magnetic source region as the strong magnetic field, and there are crescent-shaped, local, weak magnetic fields on the left and right sides. The central region corresponds to the magnetic source itself, where the magnetic field line density is the highest and the magnetic field strength is the largest; as the distance increases, the magnetic field gradually weakens toward the surroundings. The crescent-shaped local weak magnetic fields on the left and right sides are formed because, when the magnetic field lines start from the N pole and detour back to the S pole, they are concentrated on the front and rear edges, while the magnetic flux density at the center y is relatively small.

The central region of the $B_{x}$ signal is a positive strong magnetic field, and the two sides are negative weak magnetic fields. All the main paths of the magnetic field lines starting from the N pole and converging to the S pole pass through the central region, and the magnetic field lines in this region are along the positive x-axis direction, thus presenting a strong positive magnetic field. The negative weak magnetic fields on the left and right sides correspond to the magnetic pole edges of the magnet: the magnetic field lines diverge at the N pole on the left and converge at the S pole on the right. The magnetic field lines in the edge regions of both are distributed along the negative x-axis direction and only include sparse magnetic field lines at the edges, hence showing negative weak magnetic fields.

The $B_{y}$ signal exhibits a diagonal symmetric distribution. When the magnetic field lines detour from the N pole to the S pole, the magnetic field lines in the upper half of the N pole extend along the positive y direction, and those in the lower half extend along the negative y direction; while the magnetic field lines in the upper half of the S pole converge along the negative y direction, and those in the lower half converge along the positive y direction. Due to the mirror consistency of the magnetic field distribution in the upper and lower regions of the N and S magnetic poles, the $B_{y}$ signal also exhibits a diagonal symmetric distribution.

The $B_{z}$ signal is mainly concentrated in the near field of the magnetic source, with a strong positive magnetic field on the left side, a strong negative magnetic field on the right side, and an extremely weak magnetic field in the non-magnetic pole region. The left side of the magnetic source is the N pole, and the right side is the S pole. Magnetic field lines in the magnetic pole body region are highly concentrated; thus, strong magnetic fields are exhibited in the two magnetic pole regions. For the non-magnetic pole region, magnetic field lines mainly complete the detour from the diverging N pole to the converging S pole along the horizontal direction, and the proportion of the z-direction magnetic flux density component is low. Therefore, the signal amplitude in the non-magnetic pole region is close to zero.

In MFL detection signals, the axial signal $B_{x}$ and radial signal $B_{z}$ are highly sensitive to changes in key parameters such as defect length, depth, and burial depth, thus effectively reflecting defect characteristics. In contrast, the circumferential component has a small amplitude and a weak response to detection characteristic parameters; moreover, its signal features are easily submerged by noise, and collection and analysis of this component will increase system complexity. Therefore, when exploring the variation law of magnetic field signals with characteristic parameters, the analysis mainly focuses on the axial signal $B_{x}$ and the radial signal $B_{z}$.

### 3.4. Axial Signal Characteristics

**Figure 10:**
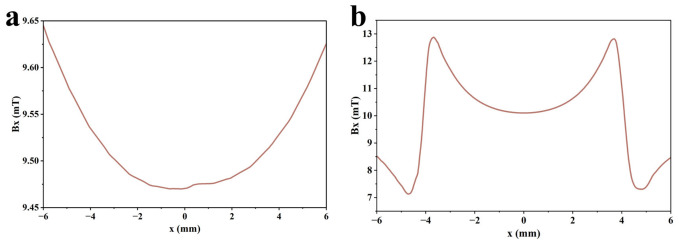
Axial signal distribution diagram: (**a**) without defects, (**b**) with defects.

In MFL testing, the axial signal is the magnetic flux density component parallel to the surface of the ferromagnetic component. It has extremely high sensitivity to defect length and serves as the core basis for quantitative evaluation of defect length [37,38]. The axial detection signals without defects and with defects with standard characteristic parameters are shown in Figure 10.

Within the approximately −8 mm to 8 mm detection range of the Hall sensor, only the permanent magnetic field signal is collected by the sensor when there is no defect. According to the distribution law of magnetic scalar potential, the axial signals generated by the N and S poles at the left and right ends of the magnetizer are both positive. When the detection position moves from the leftmost side of the sensor to the center, the distance from the N pole increases, leading to attenuation of the axial signal contributed by the N pole; meanwhile, the distance from the S pole decreases, resulting in enhancement of the axial signal contributed by the S pole. However, the attenuation amplitude of the N-pole signal is larger than the enhancement amplitude of the S-pole signal, so the axial signal shows a monotonically decreasing trend and reaches a minimum value at the center of the sensor. When the detection position moves from the center to the rightmost side, the attenuation amplitude of the N-pole signal is smaller than the enhancement amplitude of the S-pole signal, and the axial signal gradually rises.

When there is a defect, the sensor synchronously collects the defect’s leakage magnetic field signal, and the axial signals contributed by the local N and S poles formed at the defect are also positive. In the left region of the detection range, when the detection position is far from the defect, the leakage magnetic field signal is weak, and the detection signal mainly reflects the decreasing characteristic of the permanent magnetic field. As the detection position approaches the defect, the leakage magnetic field signal gradually enhances, while the permanent magnetic field signal continues to attenuate. When the leakage magnetic field signal dominates, the detection signal begins to reflect the variation law of the leakage magnetic field; the closer the detection position is to the defect, the more the axial signal rises as it approaches the local N pole.

When the detection position moves from the left edge of the defect to the center, the distance from the local N pole increases, resulting in signal attenuation; meanwhile, the distance from the local S pole decreases, leading to the enhancement of the S-pole signal. However, the attenuation effect of the N-pole signal is stronger than the enhancement effect of the S-pole signal, so the axial signal gradually decreases and forms a maximum value at the left edge of the defect. When the detection position moves from the defect center to the local S pole, the enhancement effect of the S-pole signal exceeds the attenuation effect of the N-pole signal, so the axial signal rises and forms a minimum value at the defect center. When the detection position continues to move rightward from the local S pole, the distances from both the local N and S poles increase, causing the attenuation of the leakage magnetic field signal; at the same time, the distance from the S pole on the right side of the magnetizer decreases, resulting in the enhancement of the permanent magnetic field signal. However, the leakage magnetic field signal still dominates at this time, so the axial signal decreases and forms a maximum value at the right edge of the defect. When the detection position continues to move rightward until the permanent magnetic field signal dominates, the axial signal gradually rises. Quantitative determination of defect length can be achieved by measuring the distance between the two maximum values of the axial signal.

### 3.5. Radial Signal Characteristics

**Figure 11:**
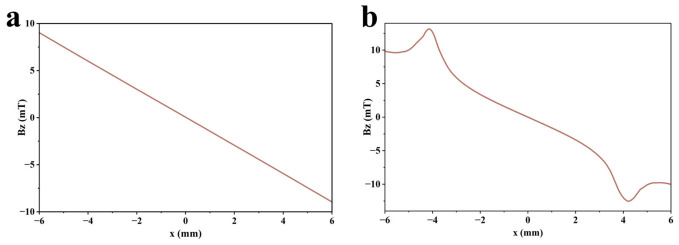
Radial signal distribution diagram: (**a**) without defects, (**b**) with defects.

In MFL testing, the radial signal is the magnetic flux density component perpendicular to the surface of the component. It has extremely high sensitivity to defect burial depth and serves as the key basis for quantitative evaluation of defect burial depth [37,38]. The radial detection signals without defects and with defects with standard characteristic parameters are shown in Figure 11.

According to the distribution law of magnetic scalar potential, when there is no defect, the radial signal contributed by the N pole on the left side of the magnetizer is positive, and the radial signal contributed by the S pole on the right side is negative. In the coordinate diagram, the detection signal shows a monotonically decreasing trend from left to right, where positive and negative values indicate the direction of the magnetic field rather than the signal intensity. When there is a defect, the radial signal contributed by the local N pole at the defect is positive, and the radial signal contributed by the local S pole at the defect is negative. When the detection position approaches the left edge of the defect from the left side, the distance from the local N pole decreases, and the radial signal is significantly enhanced, forming a maximum value at the left edge of the defect. When the detection position moves from the left edge of the defect to the right side, the distance from the local N pole increases, while the distance from the local S pole decreases, and the intensity of the negative radial signal continues to increase, forming a minimum value at the right edge of the defect. When the detection position continues to move rightward from the right edge of the defect, the distance from the local magnetic field increases, leading to attenuation of the leakage magnetic field signal; meanwhile, the distance from the S pole on the right side of the magnetizer decreases, resulting in enhancement of the permanent magnetic field signal. In the initial small range, the attenuation effect of the leakage magnetic field is stronger than the enhancement effect of the permanent magnetic field, so the radial signal rises slowly; in the subsequent stage, the enhancement effect of the permanent magnetic field dominates, and the radial signal shows a decreasing trend again. Quantitative evaluation of defect burial depth can be achieved by analyzing the variation law of the peak value of the radial signal.

### 3.6. Dynamic Scanning Detection of Defect Signals

In MFL testing, clarifying the defect position is the basis for further testing and maintenance of the specimen. The leakage magnetic field excited by the defect exhibits distance attenuation in air. When the relative distance between the defect and the sensor changes, the peak value and spatial distribution characteristics of the detection signal also change: the smaller the relative distance between the defect and the Hall sensor, the greater the magnetic induction intensity of the leakage magnetic field at the sensor, and the larger the peak value of the detection signal. In addition, the radial signal is more sensitive to changes in the relative distance in the x-direction between the sensor and the defect than the axial signal. Therefore, as the sensor gradually approaches the defect, the radial signal amplitude increases more quickly than that of the axial signal.

**Figure 12:**
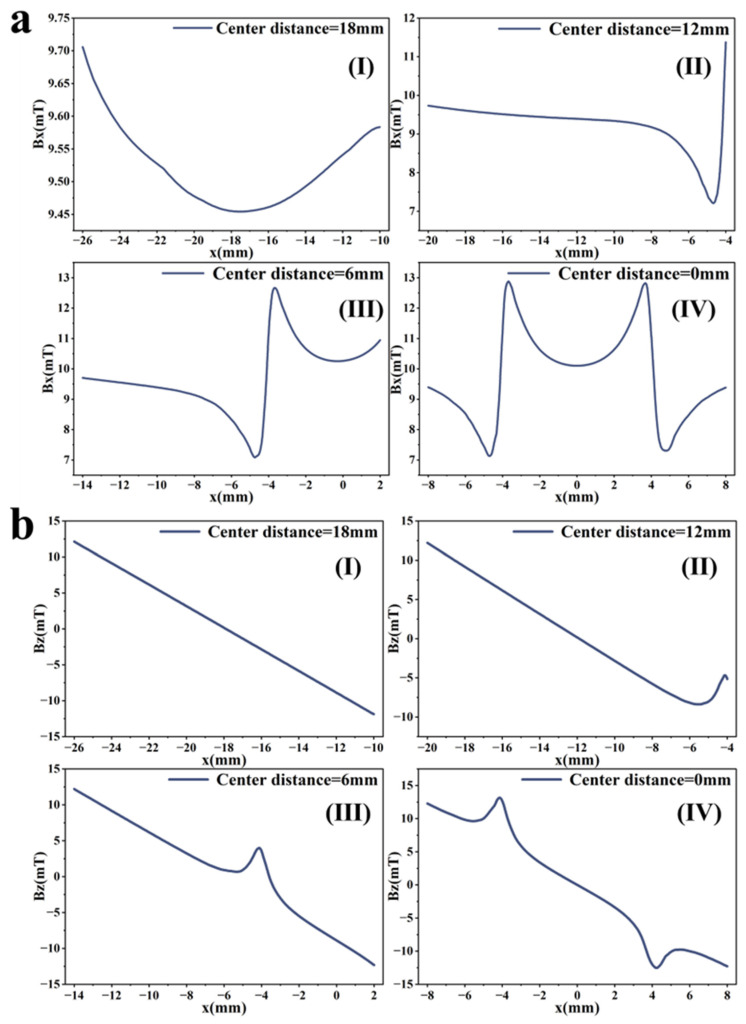
Dynamic scanning detection signal distribution diagram: (**a**) axial signal variation trend, (**b**) radial signal variation trend.

**Figure 13:**
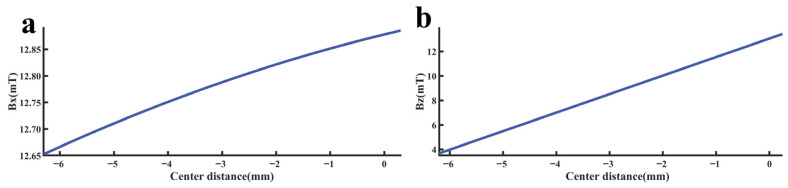
Dynamic scanning detection signal peak variation diagram: (**a**) without defects, (**b**) with defects.

To rapidly and accurately determine defect location, a dynamic scanning model is established in COMSOL for an MFL detector moving from left to right over a defect with standard parameters. By simulating the process of the detector approaching the defect from left to right, detection signals are collected at various relative distances between the sensor center and the defect center. The distribution characteristics and peak variation laws of the signals are then analyzed. Meanwhile, various function fittings are performed between the relative distance and the peak value of detection signals, and the adjusted coefficient of determination ($R_{adjusted}^{2}$) is introduced to evaluate the fitting effect. $R_{adjusted}^{2}$ is a modified index of the coefficient of determination $R^{2}$, which evaluates the goodness of fit more objectively in multiple regression by considering both the number of independent variables and the sample size. The closer the value of $R_{adjusted}^{2}$ is to 1, the better the model fitting effect and the more accurate the quantitative correlation between them. Through the fitting model, quantitative judgment of defect location can be realized. In addition, the ASTM E3023-21 standard specifies a probabilistic detection statistical analysis method based on continuous signal responses and defect size data. In model validation, $R_{2}\geq 0.7$ for linear regression models is clearly defined as the criterion for an effective model [39]. Therefore, when performing various function fittings, a fitting model is considered acceptable when $R_{adjusted}^{2}\geq 0.7$. On this basis, the fitting function with the maximum $R_{adjusted}^{2}$ is selected to obtain the optimal fitting model. The signal distribution diagram of the Hall sensor approaching the defect from left to right is shown in Figure 12, and the variation in detection signal peaks at different relative distances between the defect center and the sensor center is presented in Figure 13.

**Figure 14:**
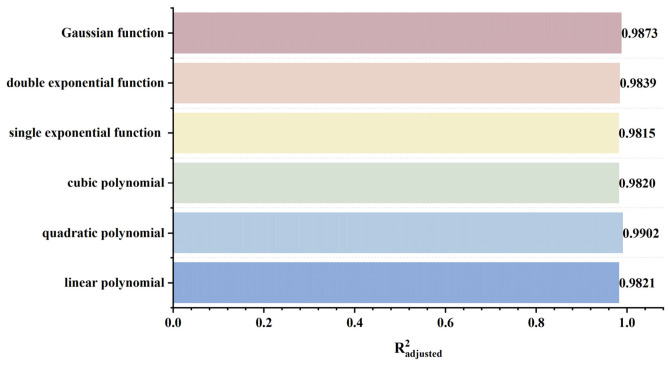
Function fitting results of the axial signal peak with dynamic scanning.

**Figure 15:**
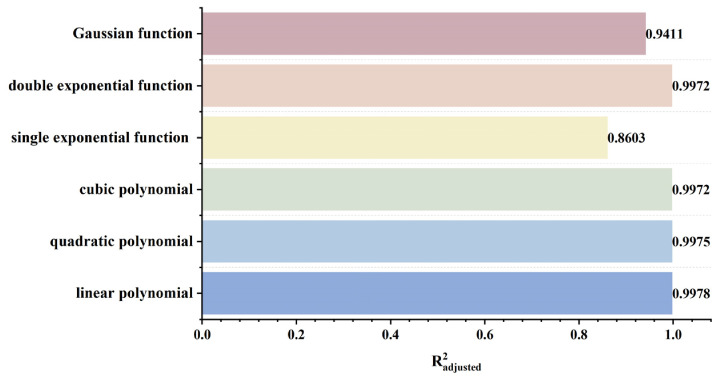
Function fitting results of the radial signal peak with dynamic scanning.

As shown in Figure 12, during a left-to-right scan, the detector first captures the leakage field signal corresponding to the local N pole of the defect. As the scan progresses, the signal from the local S pole gradually intensifies. When the detector center is positioned directly above the defect center, the detection signal exhibits left-right symmetry. As shown in Figure 13, as the relative distance between the detector and the defect decreases, the magnetic flux density of the leakage field gradually increases, leading to a corresponding rise in the detection signal peak. In addition, the radial signal exhibits a greater increase in peak amplitude than the axial signal, which is consistent with theoretical analysis. The fitting results of the detection signal peaks under different functions are shown in Figure 14 and Figure 15 ($R_{adjusted}^{2}\geq 0.7$). The optimal peak characterization model for the dynamically scanned signals obtained by fitting is as follows:

(4) $$B_{x}=-0.02x^{2}+0.02x+12.88$$

(5) $$B_{z}=1.15x+13.05$$

### 3.7. Influence of Lift-Off Value on Detection Signals

In MFL testing systems, the lift-off value refers to the vertical distance between the sensor probe and the specimen surface, and a key parameter affecting detection signal intensity. An increase in the lift-off value enhances the diffusion effect and energy attenuation of the detection signal in space, makes the spatial distribution of the magnetic field more scattered, changes the distribution characteristics of the signal, and reduces the peak value of the detection signal. Designing experiments with varying lift-off values can determine the optimal detection position of the Hall sensor; in addition, clarifying the influence of the lift-off value on detection signals provides a basis for correcting detection parameter errors and improves the accuracy of defect detection.

**Figure 16:**
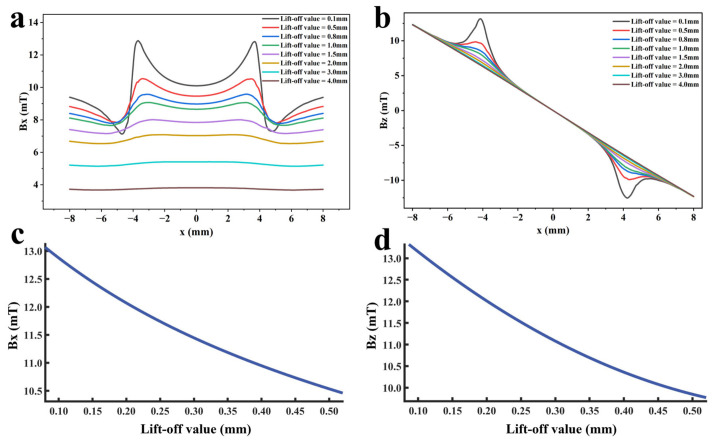
The effect of lift-off value on detection signals: (**a**) axial detection signal distribution diagram, (**b**) radial detection signal distribution diagram, (**c**) variation in axial detection signal peak, (**d**) variation in radial detection signal peak.

To investigate the influence of lift-off value variation on detection signals, the sensor is placed directly above the defect, and other defect parameters are kept at standard values. Within the range of 0.1 mm to 4 mm, different lift-off values are set to ensure that the variation in detection signals is only caused by the change of lift-off value. Furthermore, multiple function fittings between lift-off value and detection signal peak were performed to achieve quantitative determination of lift-off value. A finite element model is established based on the above parameters. The detection signal distribution diagrams corresponding to different lift-off values obtained through simulation calculation are shown in Figure 16a,b, and the extracted variation of detection signal peaks with lift-off values is shown in Figure 16c,d.

**Figure 17:**
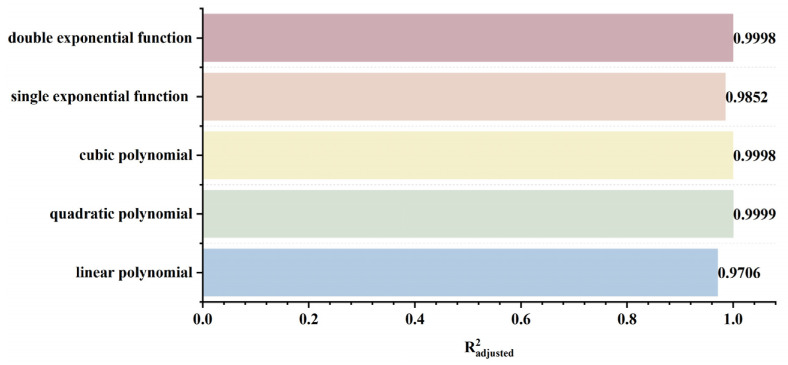
Function fitting results of the axial signal peak with varying lift-off values.

**Figure 18:**
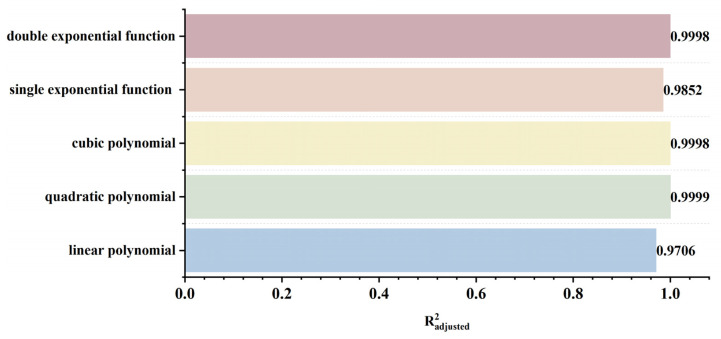
Function fitting results of the radial signal peak with varying lift-off values.

As shown in Figure 16, when the sensor lift-off is 0.1 mm, the detection signals exhibit the most pronounced characteristics. Accordingly, all subsequent experiments were conducted at a lift-off of 0.1 mm. For the axial signal, an increase in lift-off significantly enhances the spatial diffusion of the magnetic field, thereby reducing the difference in detection signals between the defect edge and its center. Moreover, the independent magnetic fields generated by the equivalent magnetic charges on both sides of the defect overlap and merge during propagation. As a result, the original double-peak feature—corresponding to the two ends of the defect—gradually diminishes, and the axial signal transitions from a double-peak waveform to a smooth single-peak waveform. As lift-off increases, the peak value of the radial signal gradually decreases. When the lift-off becomes sufficiently large such that the received leakage field signal is extremely weak, the magnitude of the radial detection signal approaches that of the permanent magnetic field. Overall, the peak values of both the axial and radial signals exhibit a uniform and gradual decline with increasing lift-off. The fitting results of the detection signal peaks with different functions are shown in Figure 17 and Figure 18 ($R_{adjusted}^{2}\geq 0.7$). The optimal characterization model describing the influence of sensor lift-off on the detection signal peak obtained by fitting is as follows:

(6) $$B_{x}=2.43e^{-3.98x}+11.52e^{-0.24x}$$

(7) $$B_{z}=10.47x^{2}-14.54x+14.50$$

### 3.8. Influence of Defect Burial Depth on Detection Signals

In MFL testing, defect burial depth is a key parameter that determines the distribution characteristics of detection signals. The larger the defect burial depth, the greater the magnetic circuit space available inside the specimen for accommodating magnetic field distortion. More paths for magnetic lines of force to bypass the defect region are constrained within the specimen, thereby reducing the number of magnetic lines of force squeezed into the air, changing the characteristics of detection signals and leading to an attenuation trend in signal peak values [40,41,42]. In actual testing, if the influence of defect burial depth is ignored, defect size and other parameters may be misjudged, resulting in deviations in structural safety assessment results. Therefore, analyzing the influence of defect burial depth on detection signals can improve the reliability and accuracy of MFL testing technology.

**Figure 19:**
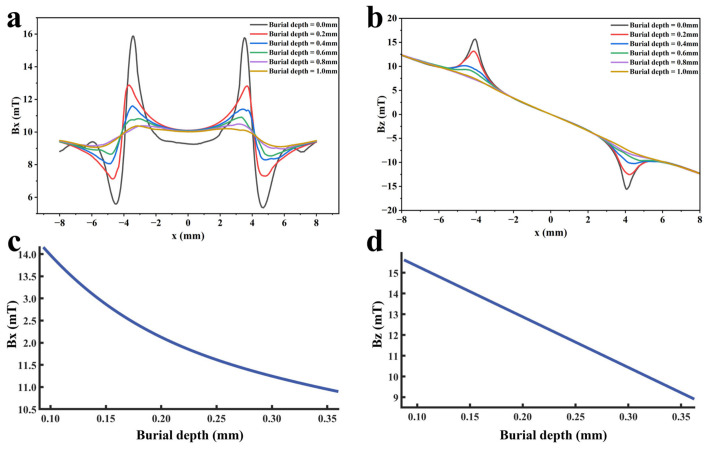
The effect of defect burial depth on detection signals: (**a**) axial detection signal distribution diagram, (**b**) radial detection signal distribution diagram, (**c**) variation in axial detection signal peak, (**d**) variation in radial detection signal peak.

To investigate the influence of defect burial depth on detection signals, other defect parameters are kept at standard values. Within the range of 0 mm to 1.0 mm, different burial depth values are set with a step size of 0.2 mm to ensure that the variation in detection signals is only affected by defect burial depth. A finite element model is established, and multiple function fittings are performed between the defect burial depth and the detection signal peak to realize quantitative determination of the defect burial depth. The detection signal distributions obtained through simulation calculations are shown in Figure 19a,b. The extracted variation in detection signal peaks with defect burial depth is shown in Figure 19c,d.

**Figure 20:**
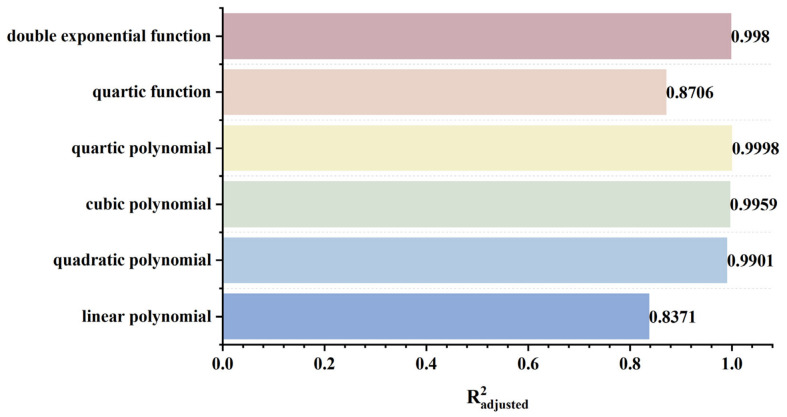
Function fitting results of the axial signal peak at varying defect burial depths.

**Figure 21:**
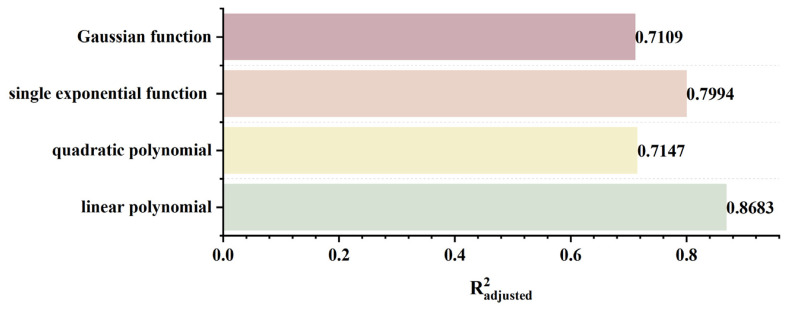
Function fitting results of the radial signal peak at varying defect burial depths.

As shown in Figure 19, the peak values of both the axial and radial signals decrease as the defect burial depth increases. At shallow burial depths, the axial signal exhibits a clear double-peak characteristic. As the burial depth increases, the leakage magnetic field signal is progressively attenuated, and the amplitude difference between the double peaks at the defect edges and the valley at the defect center diminishes. In addition, the increased spatial diffusion of the leakage magnetic field enhances the field diffusion effect, causing the originally separated double magnetic charge fields to overlap and merge during propagation. Consequently, the double-peak feature of the axial signal gradually weakens and transitions into a broad single-peak waveform. Furthermore, when the burial depth is shallow, the leakage magnetic field signal is strong, and variations in burial depth have a pronounced effect on the axial signal intensity, resulting in a rapid decrease in peak value. At greater burial depths, the leakage field signal becomes weak, and further increases in burial depth exert only a slight influence on the axial signal intensity, leading to a slow decline in peak value. The peak value of the radial signal also decreases with increasing defect burial depth. Owing to the relatively uniform spatial distribution of the radial field, its peak value exhibits a linear decrease as burial depth increases. The fitting results of the detection signal peaks with different functions are shown in Figure 20 and Figure 21 ($R_{adjusted}^{2}\geq 0.7$). The optimal characterization model describing the influence of defect burial depth on the detection signal peak obtained by fitting is as follows:

(8) $$B_{x}=13.35x^{4}-34.69x^{3}+35.64x^{2}-19.54x+15.61$$

(9) $$B_{z}=-12.20x+16.53$$

### 3.9. Influence of Defect Length on Detection Signals

As a core geometric parameter characterizing the degree of specimen damage, defect length is directly related to the subsequent specimen safety assessment and maintenance decision-making. When the defect length changes, the spatial distribution positions of the equivalent N and S magnetic charges in the defect region are correspondingly altered, thereby leading to differences in the distribution positions of the peak and valley values of detection signals [41,42,43]. With an increase in defect length, the magnetic field leaking from the left edge of the defect to the air needs to pass through a longer magnetic circuit path when returning to the right edge; meanwhile, the diffusion space of the leakage magnetic field in the air is also increased accordingly. These two effects reduce the magnetic flux density of the leakage magnetic field, thus decreasing the peak value of the detection signal.

**Figure 22:**
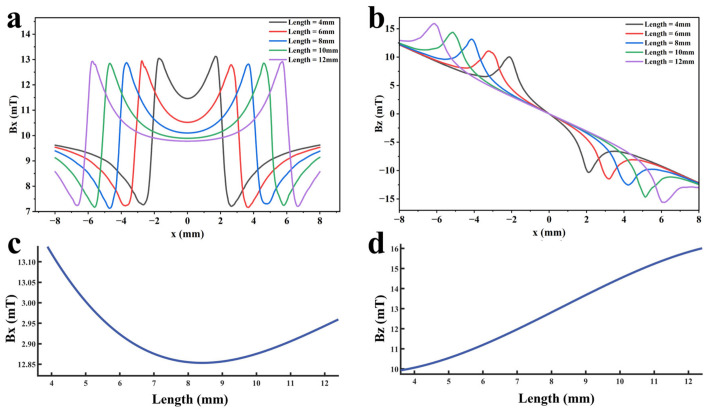
The effect of defect length on detection signals: (**a**) axial detection signal distribution diagram, (**b**) radial detection signal distribution diagram, (**c**) variation in axial detection signal peak, (**d**) variation in radial detection signal peak.

To explore the variation law of detection signals with respect to defect length, a finite element model was established and calculated with all other parameters maintained at standard values. Defect lengths were set to 2.0 mm, 2.5 mm, 3.0 mm, 3.5 mm, and 4.0 mm to ensure that signal variations were caused solely by length changes. Furthermore, multiple function fittings are performed between the defect length and the detection signal peak to quantify the defect length. The resulting signal distributions are shown in Figure 22a,b. Figure 22c,d shows the variation in signal peaks with defect length.

**Figure 23:**
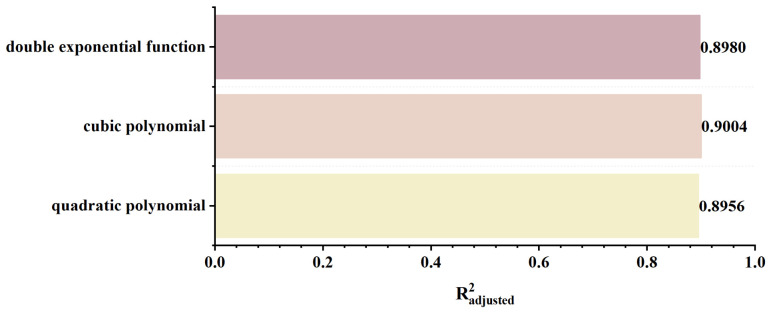
Function fitting results of the axial signal peak at varying defect lengths.

**Figure 24:**
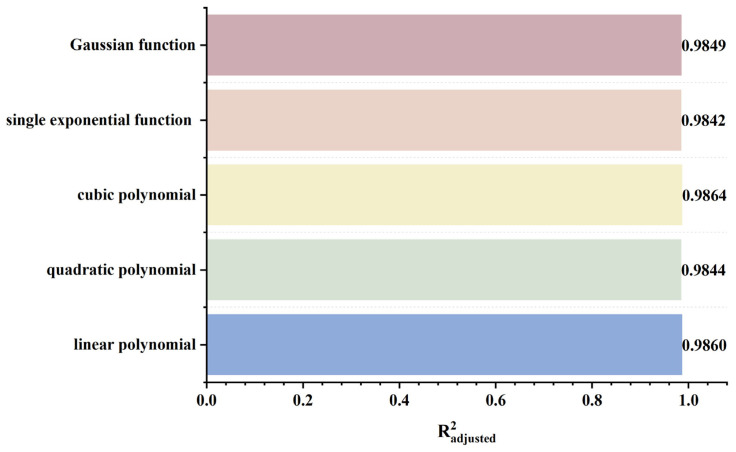
Function fitting results of the radial signal peak at varying defect lengths.

As shown in Figure 22, as the defect length increases, the separation between the two peaks of the axial signal widens correspondingly, as does the peak-to-valley interval of the radial signal. However, their peak amplitudes exhibit distinctly different trends: the axial signal peak first decreases and then increases, whereas the radial signal peak increases monotonically. This behavior arises from two competing mechanisms. First, an increase in defect length reduces the leakage field intensity due to elongation of the magnetic circuit and enhanced field diffusion. Second, the defect edges move closer to the detector’s N or S poles, thereby strengthening the background magnetic field. For the axial signal, at small defect lengths, the attenuation of the leakage field outweighs the enhancement of the background field, leading to an initial decrease in the peak value. As the defect length continues to increase, this imbalance diminishes and eventually reverses, resulting in a subsequent increase in the peak. For the radial signal, the enhancement of the background field consistently dominates the attenuation of the leakage field, producing a monotonic increase in the peak value. The fitting results of the detection signal peaks under different functions are shown in Figure 23 and Figure 24 ($R_{adjusted}^{2}\geq 0.7$). The optimal characterization model describing the influence of defect length on the detection signal peaks obtained by fitting is as follows:

(10) $$B_{x}=-8.00\times 10^{-4}x^{3}+0.03x^{2}-0.35x+14.06$$

(11) $$B_{z}=-8.80\times 10^{-3}x^{3}+0.22x^{2}-0.95x+10.94$$

### 3.10. Influence of Defect Width on Detection Signals

Defect width is a key geometric parameter for assessing the reliability of specimens and directly affects the prediction of the residual service life of specimens. As the defect width increases, the distribution range of magnetic charges along the width direction at the defect becomes wider, resulting in a stronger leakage magnetic field, while the background magnetic field intensity remains unchanged. Therefore, the peak value of the detection signal increases with increased defect width. The radial signal originates from the distribution of magnetic charges perpendicular to the material surface, and the field lines diffuse rapidly to the surroundings. The axial signal originates from the distribution of magnetic charges along the magnetization direction, and the field lines tend to extend along the material surface. Therefore, the radial signal is more sensitive to changes in width, resulting in greater variation in signal peak value [44,45].

**Figure 25:**
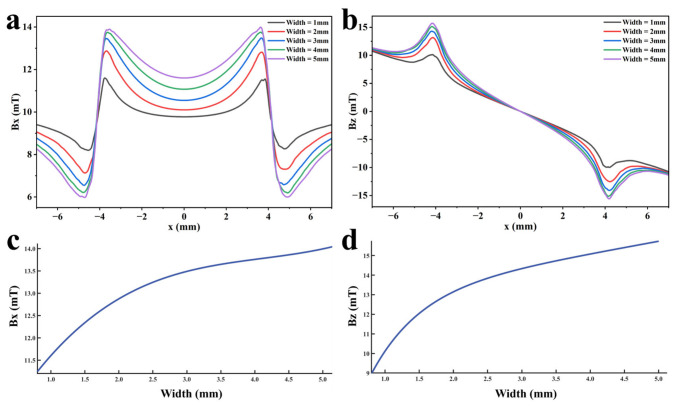
The effect of defect width on detection signals: (**a**) axial detection signal distribution diagram, (**b**) radial detection signal distribution diagram, (**c**) variation in axial detection signal peak, (**d**) variation in radial detection signal peak.

To explore the influence law of defect width on detection signals, with other defect parameters set to standard values, defect widths of 1 mm, 2 mm, 3 mm, 4 mm, and 5 mm are set, ensuring that the detection signal is only affected by the change in defect width. Various function fittings are performed between the defect width and the peak value of the detection signal to quantify defect width. The detection signal distribution obtained by calculating the established finite element model is shown in Figure 25a,b, and the variation in the detection signal peak value with the change in defect length is shown in Figure 25c,d.

**Figure 26:**
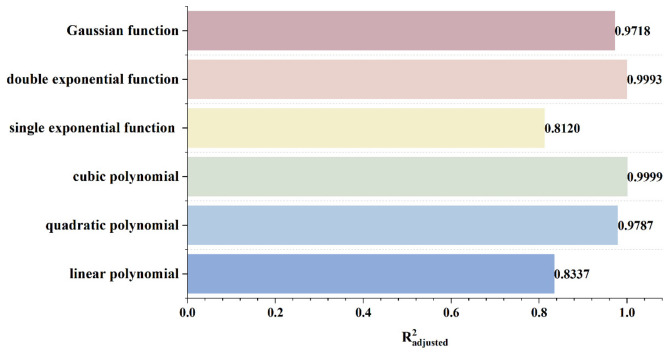
Function fitting results of the axial signal peak with varying defect widths.

**Figure 27:**
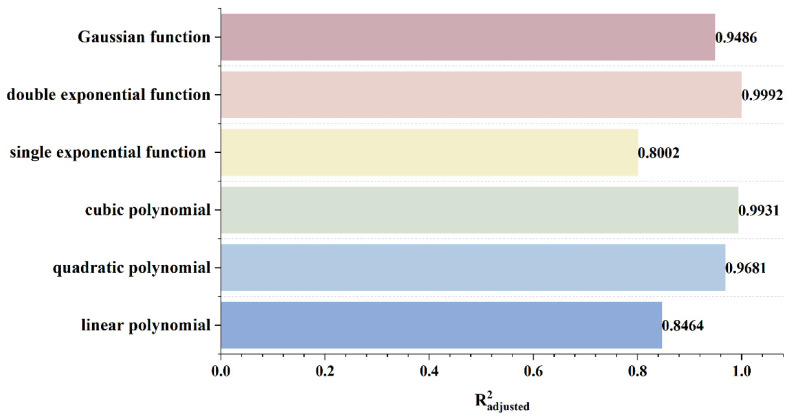
Function fitting results of the radial signal peak with varying defect widths.

It can be seen from Figure 25 that, when the defect width increases, the peak values of both the axial signal and the radial signal show an upward trend, but the spatial distribution characteristics of the signals remain basically unchanged. Defect width only affects the signal intensity, not the signal distribution. In addition, compared with the radial signal, the peak value of the axial signal increases at a slower rate. The rising rates of both the axial signal and the radial signal decrease with the increase of defect width. As the defect width increases, the spatial distribution range of the leakage magnetic field expands, and the influence of the change in leakage magnetic field intensity caused by the increase in width on the entire leakage magnetic space decreases. Therefore, the rising rate of the detection signal decreases. The fitting results of the detection signal peak values with different functions are shown in Figure 26 and Figure 27, and the optimal characterization model of the defect depth on the detection signal peak value obtained by fitting is

(12) $$B_{x}=0.05x^{3}-0.64x^{2}+2.83x+9.36$$

(13) $$B_{z}=12.90e^{0.04x}-13.30e^{-1.39x}$$

### 3.11. Influence of Defect Depth on Detection Signals

Defect depth is a key parameter that characterizes the severity of specimen damage, as it directly determines the specimen’s load-bearing capacity and remaining service life. Accurate evaluation of depth is therefore of great significance for safe service. An increase in defect depth raises both the amount and the density of magnetic charge at the defect interface, thereby strengthening the leakage magnetic field signal and intensifying the detection signal [46,47]. Moreover, the radial signal exhibits higher sensitivity to depth variations than the axial signal; consequently, depth changes result in larger fluctuation of the amplitude of the radial signal peak.

**Figure 28:**
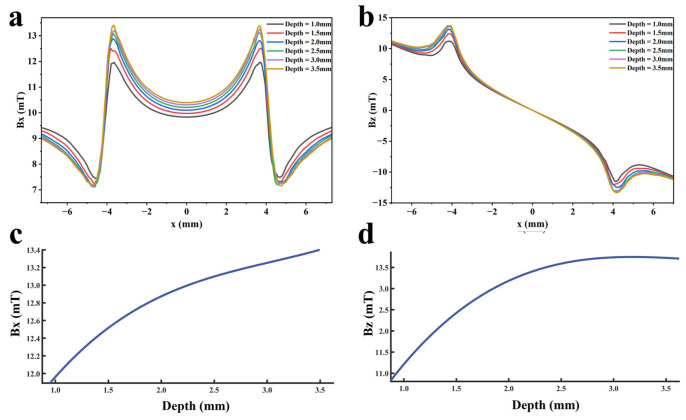
The effect of defect depth on detection signals: (**a**) axial detection signal distribution diagram, (**b**) radial detection signal distribution diagram, (**c**) variation in axial detection signal peak, (**d**) variation in radial detection signal peak.

To quantify the influence of defect depth on detection signals, based on the premise that other parameters are fixed at standard values, defects with different depths are set with a step size of 0.5 mm within the range of 1.0 mm to 3.5 mm to ensure that the detection signals are only affected by the variation in defect depth. Furthermore, multiple function fittings are performed between the defect depth and the detection signal peak to quantify the defect depth. A finite element model is established and calculated, and the obtained detection signal distributions are shown in Figure 28a,b. The variation law of detection signal peaks with defect depth is shown in Figure 28c,d.

**Figure 29:**
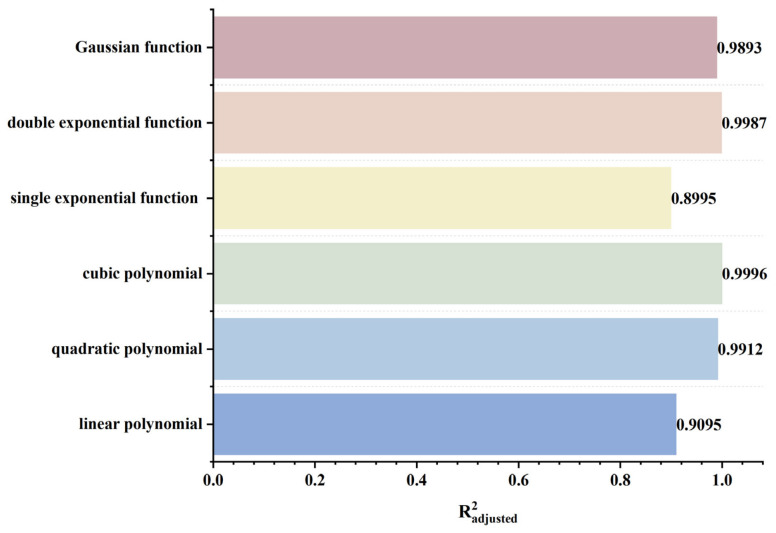
Function fitting results of the axial signal peak at varying defect depths.

**Figure 30:**
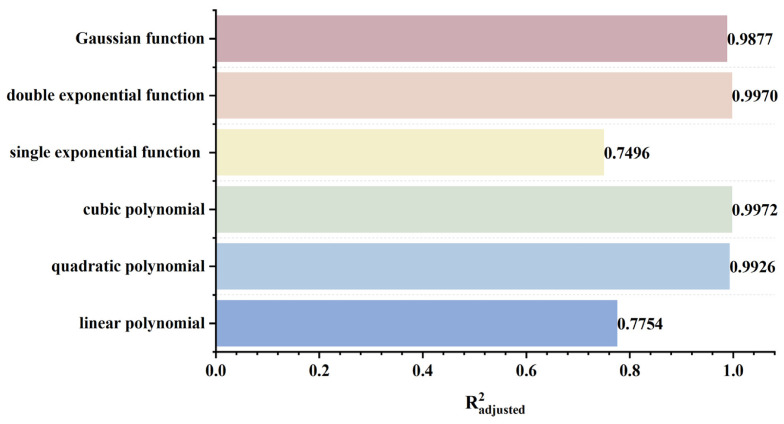
Function fitting results of the radial signal peak at varying defect depths.

As can be seen from Figure 28, with the increase of defect depth, the amplitudes of both the axial and radial signals show an upward trend, but the spatial distribution characteristics of the signals do not change—that is, the defect depth only affects the signal intensity, and not the signal distribution. Moreover, the increasing rate of the axial signal peak decreases relatively slowly with increasing defect depth, while the increasing rate of the radial signal peak decreases rapidly. When the defect depth increases, the increasing amplitude of the magnetic charge density caused by the defect gradually decreases, which leads to a decrease in the growth rate of the detection signal peak. As the defect depth increases, the added magnetic charge pairs are in the same direction in the axial direction, resulting in magnetic field superposition; whereas in the radial direction, they are in like directions, leading to magnetic field cancellation. Therefore, the decreasing rate of the axial signal peak is smaller than that of the radial signal. The fitting results of the detection signal peaks with different functions are shown in Figure 29 and Figure 30 ($R_{adjusted}^{2}\geq 0.7$). The optimal characterization model describing the influence of defect depth on the detection signal peak obtained by fitting is presented below:

(14) $$B_{x}=0.08x^{3}-0.78x^{2}+2.64x+10.01$$

(15) $$B_{z}=0.13x^{3}-1.48x^{2}+5.50x+7.06$$

### 3.12. Effect of Specimen Size on Detection Signals

**Figure 31:**
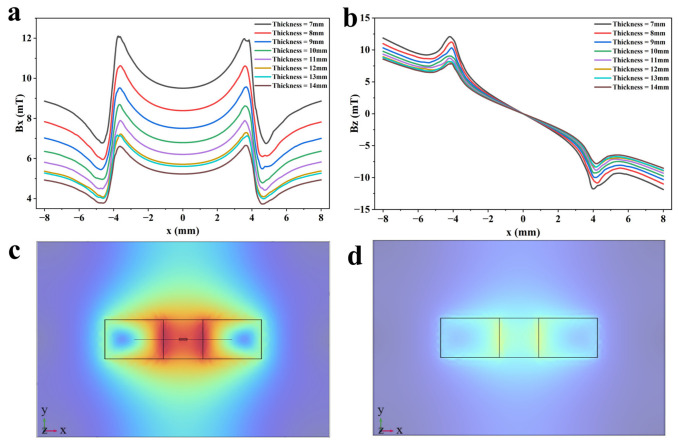
Effect of specimen size on detection signals: (**a**) axial signal distribution, (**b**) radial signal distribution, (**c**) magnetic flux density heat map at thickness = 7 mm, (**d**) magnetic flux density heat map at thickness = 14 mm. (Colors from red to blue represent a decrease in magnetic flux density).

In magnetic flux leakage testing, specimen size is one physical factor that regulates magnetization and affects signal intensity. As a key part of the magnetic circuit, specimen size directly affects the effective length and magnetic resistance of the magnetic circuit [48]. Under the same magnetization conditions, larger specimen size lengthens the internal magnetic path, increases magnetic resistance, reduces overall magnetization, and weakens the detection signal [49]. Investigating the relationship between specimen dimensions and signal strength is of great significance to the applicability and application range of magnetic flux leakage detectors. The specimen thickness was set from 7 to 14 mm, with a 1 mm step size, to study the signal intensity variation at different sizes. The detection signal distributions calculated by the finite element model are shown in Figure 31a,b. The magnetization intensities at 7 mm and 14 mm are shown in Figure 31c and Figure 31d, respectively.

It can be seen from the figure that, as specimen thickness increases, its magnetization intensity decreases significantly, leading to a downward trend in detection signal strength. The axial component drops from 12.10 mT to 7.29 mT, with a total attenuation of about 39.75%. The radial component decreases from 12.07 mT to 8.15 mT, with a total attenuation of about 32.48%. In addition, when the specimen thickness is 14 mm, the specimen cannot experience saturated magnetization, so effective detection cannot be achieved, meaning the effective detection thickness range of the detector is within 13 mm. To evaluate the detection performance of the detector at different thicknesses, statistical analysis was performed on the peak values of axial and radial signals to judge the applicability and dispersion of the detector. The peak analysis results of axial and radial signals are shown in Table 1.

It can be seen from the analysis results that the standard deviation of the axial signal is 1.79 mT, and the coefficient of variation is 19.12%, while the mean value of the radial signal is 9.92 mT, the standard deviation is 1.54 mT, and the coefficient of variation is 15.52%. The standard deviation and coefficient of variation of the axial signal are both larger than those of the radial signal, indicating that the specimen thickness has a more significant effect on the axial signal. In other words, the axial signal is more sensitive to the specimen size, while the radial signal shows higher stability at varying thickness.

### 3.13. Detection Signals of Defects with Different Shapes

**Figure 32:**
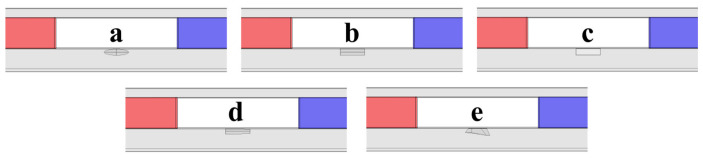
Inspection models for various defect geometries: (**a**) ellipsoidal defect, (**b**) cylindrical defect, (**c**) rectangular defect, (**d**) conical defect, (**e**) irregular defect. (The red magnet represents a positive pole on its upper surface, while the blue magnet represents a negative pole on its upper surface).

**Figure 33:**
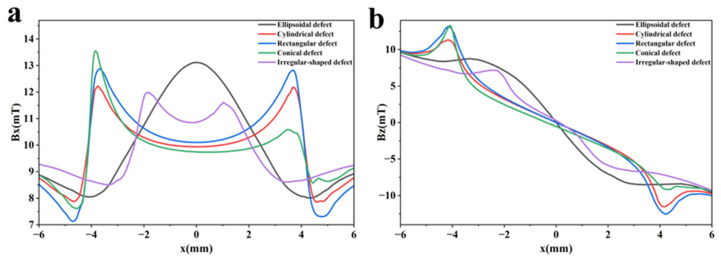
Detection signals of different defect shapes: (**a**) axial signal, (**b**) radial signal.

Defects of different shapes exist in actual detection situations. In magnetic flux leakage testing, their signals differ in terms of spatial distribution and amplitude. Identifying defects of different shapes is of great significance for the accuracy of detection results. To evaluate the ability of the established detector to discriminate defects of different shapes, five defect shapes were tested: ellipsoidal, cylindrical, rectangular, conical, and irregular. All defects have a maximum length of 8 mm and a maximum width and depth of 2 mm. The established detection model is shown in Figure 32, and the obtained detection signal distribution diagrams are shown in Figure 33.

The axial signal of an ellipsoidal defect is a smooth single peak, and the radial signal is a smooth double peak. Magnetic charges distribute continuously along the ellipsoidal defect surface, leading to smooth waveforms. The defect depth and signal amplitude increase toward the center, so the axial signal shows a single peak. The axial signals of cylindrical, rectangular, and conical defects all show double-peak features. The two edges of a conical defect have different depths, so the left and right signal peaks differ in magnitude. Cylindrical and rectangular defects show symmetric signals, but rectangular defects have higher peak values. Rectangular defects have sharp edges, while cylindrical defects have smooth, curved surfaces. Magnetic field lines distort more strongly at rectangular defects, producing larger signal peaks. The detection signals of irregular defects are usually asymmetric, with multi-peak superposition and non-uniform variation, showing complex distributions. It can be seen from the detection signal diagrams that the established detector is sensitive to changes in defect shape and can distinguish defects of different shapes.

### 3.14. Random Weak Perturbation Experiment

In actual MFL testing, the sensor lift-off value is prone to deviation during assembly and movement, which will introduce errors into detection signals. Naturally formed defects are characterized by inhomogeneity, and their burial depths may have random deviations. In addition, the relative permeability of the specimen material will also change due to temperature fluctuations, stress evolution, and other factors. To quantify the influence of the above three types of uncertainties on MFL signals, and to verify the adaptability and robustness of the model to actual working condition fluctuations, 1% random perturbations are introduced to the defect burial depth, sensor lift-off value, and relative permeability of the material, while keeping the geometric dimensions of the defect at standard values. Comparative simulation experiments are carried out to obtain the magnetic flux density B values under different perturbation conditions, and their confidence intervals, standard deviations, and coefficients of variation are calculated. The simulation results under various parameter perturbations are shown in Table 2.

Through statistical analysis, the average value of the simulation results was determined to be 16.43 mT, and the sample standard deviation is 0.195 mT, indicating that, after applying 1% random perturbations to the defect burial depth, lift-off value, and material permeability, the fluctuation amplitude of the magnetic flux density is small, which proves that the established model has good numerical stability regarding minor changes in the actual detection environment and material properties. The 95% confidence interval of the simulation results is [16.34, 16.52] mT, with a narrow interval width, and both the upper and lower limits of the interval are close to the average value, indicating that the simulation results with parameter perturbations have high statistical accuracy and yield reliable results. The calculated coefficient of variation is 1.19%, which is far lower than the general threshold of 5% in engineering simulation, indicating that the error caused by lift-off deviation, inhomogeneous defect burial depth, and material permeability change accounts for a low proportion of variation, and that the model has good robustness and applicability.

In conclusion, when facing lift-off errors, defect inhomogeneity, and material environment fluctuations in actual detection, the constructed MFL finite element model exhibits stable response, reliable results, and low model dispersion, which can effectively realize MFL defect detection in real-world scenarios.

## 4. Data Verification and Correction

In MFL testing, the double-peak distance of the axial signal is the core basis for identifying defect length, which has little correlation with the width and depth of the defect [50]. Theoretically, the local N and S poles of the leakage magnetic field correspond to the two edge positions of the defect; therefore, the distance between the double-peak positions of the axial signal should be consistent with the actual length of the defect. However, in the actual detection process, deviations between the peak positions of the simulated axial signal and the true edges of the defect are induced by multiple factors, such as the spatial diffusion effect of the magnetic field, the random burial depth of the defect, the variation of the lift-off value of the detection probe, and the fluctuation of the relative permeability of the specimen during magnetization. These deviations directly affect the accuracy of defect length evaluation.

To quantify and correct this error, the three-dimensional (3D) magnetic dipole method is adopted in this study to verify the simulation results. First, based on the geometric parameters and magnetic field characteristics of the defect, a 3D magnetic dipole model of the defect is established, and the peak distribution results of the axial leakage magnetic field signal are obtained through theoretical calculations. Then, the theoretical results are compared with those obtained from COMSOL simulations, and the accuracy of the simulation results is analyzed. Finally, a correction model is established based on the deviations to calibrate the peak positions of the simulated axial signals.

By contrasting the theoretical predictions derived from the 3D magnetic dipole approach with the outcomes of simulations, the validity of the finite element model is confirmed. Additionally, the correction process significantly mitigates the impact of various physical factors on the axial signal, leading to a decrease in the discrepancy between the peak location and the true boundary of the defect. Consequently, the precision of MFL testing for assessing defect length is enhanced. This research offers a solid data foundation for the future quantitative analysis of defect properties.

### 4.1. Quantitative Calculation of Axial Signals in a 3D Magnetic Dipole Model

**Figure 34:**
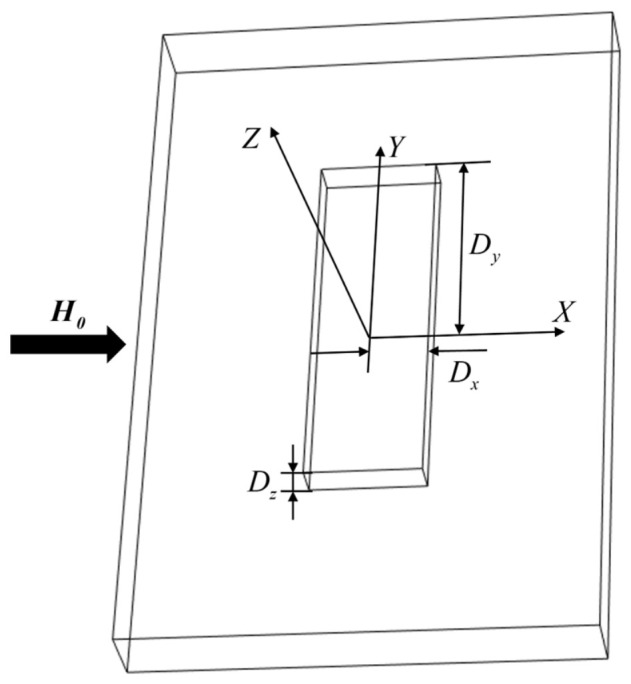
3D rectangular defect model diagram.

Taking the center point of the upper surface of the rectangular defect as the origin of coordinates, a 3D Cartesian coordinate system is established. The length, width, and height of the defect are defined as $2D_{x}$, $2D_{y}$, and $D_{z}$ respectively, and the corresponding 3D rectangular defect model is shown in Figure 34.

**Figure 35:**
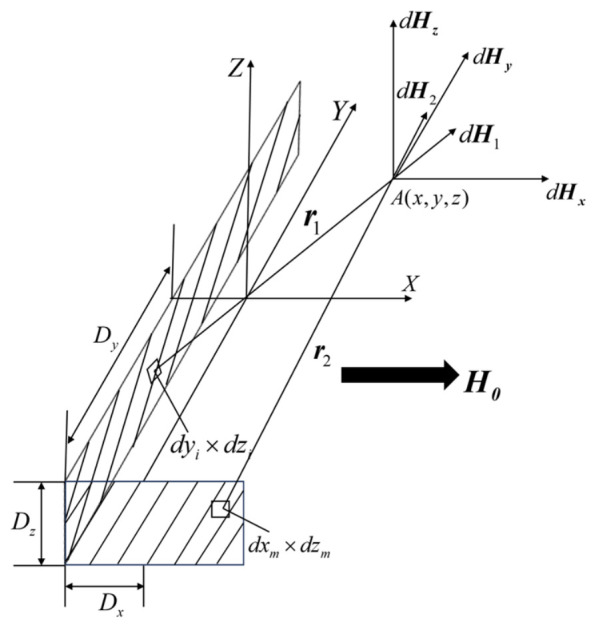
Magnetic field distribution of magnetic pole micro-surface elements on the unilateral horizontal and vertical wall surfaces of the defect.

$H_{0}$ represents the internal magnetic field of the specimen after being saturated and magnetized. It is assumed that the magnetic flux inside the specimen is uniformly distributed; at this time, the magnetic charge density on the defect wall surfaces also exhibits a uniform distribution state. The magnetic field distribution characteristics of the magnetic pole micro-surface elements on the unilateral horizontal and vertical wall surfaces of the defect are shown in Figure 35.

The coordinate of the detection point in 3D space is taken as A(x,y,z); in addition, the coordinates of the magnetic charge surface element points on the horizontal and vertical wall surfaces of the defect are defined as $\left(x_{i},y_{i},z_{i}\right)$ and $\left(x_{m},y_{m},z_{m}\right)$, respectively. Then, the magnetic field intensity generated by the micro-surface element on the magnetic charge surface at the detection point A can be expressed as

(16) $$\begin{array}{c} dH_{1}=\frac{\sigma_{1}dy_{i}dz_{i}}{2\pi \mu_{0}r^{2}}r_{1}\\ dH_{2}=\frac{\sigma_{2}dx_{m}dz_{m}}{2\pi \mu_{0}r^{2}}r_{2} \end{array}$$

where $\sigma_{1}$ and $\sigma_{2}$ are the magnetic charge surface densities of the horizontal and vertical wall surfaces of the defect, respectively. Their values can be calculated as follows:

(17) σ1=5.3DzDy+1DzμDy+1H0σ1=5.3DzDx+1DzμDx+1H0

In the formula, μ is the relative permeability of the specimen material, and $H_{0}$ is the magnitude of the magnetic field intensity at which the specimen is magnetized.

When the position coordinate of the defect’s horizontal wall surface on the x-axis is $x_{i}$, its range on the y-axis is from $-D_{y}$ to $D_{y}$, and its range on the z-axis is from 0 to $-D_{z}$; the position coordinate of the defect’s vertical wall surface on the y-axis is $y_{m}$, its range on the x-axis is from $-D_{x}$ to $D_{x}$, and its range on the z-axis is from 0 to $-D_{z}$. According to the defect wall surface model shown in Figure 19, two-dimensional integration is performed on the micro-surface elements of the two wall surfaces, and the magnitudes of the axial leakage magnetic fields formed by the two defect wall surfaces at the detection point A(x,y,z) are obtained as follows:

(18) H1xi=σ14π∫−Dz0∫−DyDyxdyidzix−xi2+y−yi2+z−zi232H2ym=σ24π∫−Dz0∫−DxDxydxmdzmx−xm2+y−ym2+z−zm232

For a rectangular defect, the total horizontal wall axial leakage magnetic field generated at the detection point A(x,y,z) is actually the combined magnetic field of the positive magnetic dipole at $x_{i}=-D_{x}$ and the negative magnetic dipole at $x_{i}=D_{x}$. The total vertical wall axial magnetic field is the combination of the positive magnetic dipole at $y_{m}=-D_{y}$ and the negative magnetic dipole at $y_{m}=D_{y}$. Therefore, the total axial leakage magnetic field $H_{x}$ of the defect wall surface at A is expressed as

(19) $$H_{x}=\left[H_{1}\left(-D_{x}\right)-H_{1}\left(D_{x}\right)\right]•e_{x}+\left[H_{2}\left(-D_{y}\right)-H_{2}\left(D_{y}\right)\right]•e_{x}$$

where $e_{x}$ is the unit vector in the x--axis direction.

### 4.2. Data Verification

To verify the accuracy of the simulation results, the defect height is designed as $D_{z}=2\;mm$, the defect width as ${2D}_{y}=2\;mm$, and the coordinate of the detection point A as the central position (0, 0, 0.1) of the Hall sensor when the lift-off value is 0.1 mm. The relative permeability of the specimen is 300. Defect lengths are set to 4 mm, 6 mm, 8 mm, 10 mm, and 12 mm. First-order and second-order derivatives of the axial signal $H_{x}$ are calculated via MATLAB R 2024 a. The x-coordinate corresponding to the position where the first derivative of $H_{x}$ is 0 and the second derivative is less than 0 is the peak position of $H_{x}$. The distance between the x-coordinates corresponding to the two peaks is the theoretically calculated defect length. The theoretical defect length obtained by derivative calculation based on the 3D magnetic dipole method is compared with the defect length obtained from simulations to verify the simulation results. The theoretical and simulation data are shown in Table 3.

The data presented in Table 3 reveal that the discrepancies between the COMSOL simulation results and the theoretical calculations using the 3D magnetic dipole method do not exceed 0.17 mm, with relative errors remaining below 3.00%. The average relative error is 1.29%, and the standard deviation is 0.87%, indicating that the finite element simulation model established in this study is consistent with the theoretical values and exhibits high accuracy and stability. The errors may be related to factors such as the precision of mesh division in the COMSOL simulation, and these errors are within an acceptable range.

### 4.3. Data Correction

In MFL testing, defect length is typically characterized by the distance between the double peaks of the axial leakage magnetic field signal $H_{x}$. Ideally, these peak positions should correspond to the two edges of the defect, with their separation equaling the actual defect length. However, due to factors such as magnetic field diffusion and sensor lift-off, errors arise when calculating defect length directly from the signal peak positions. Therefore, based on the theoretical framework of the 3D magnetic dipole method, a signal correction approach using high-order derivative analysis of $H_{x}$ is proposed to improve the accuracy of defect length identification.

**Figure 36:**
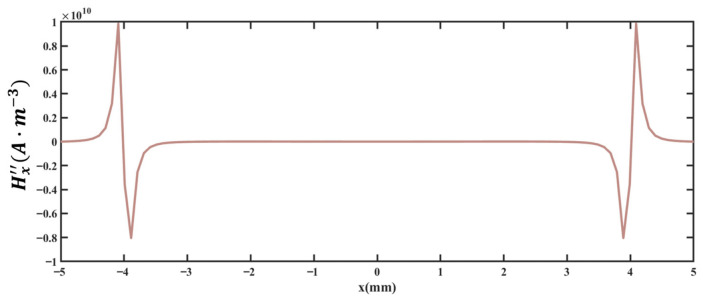
$H_{x}^{\prime \prime }$ with respect to x.

The second-order derivative of the axial signal $H_{x}$ is computed, and the distribution characteristics of $H^{\prime \prime }$ with respect to the spatial coordinate x are shown in Figure 36. It can be seen that, within both the positive and negative intervals of x, there exists a set of “maximum-minimum” characteristic pairs in $H^{\prime \prime }$. Based on this pattern, we propose to correct the original peak position of $H_{x}$ to the midpoint between the positions of the maximum and minimum values of $H^{\prime \prime }$ in the corresponding interval. This correction can compensate for the peak offset caused by magnetic field diffusion and other factors, bringing the corrected peak position closer to the actual edge of the defect.

**Figure 37:**
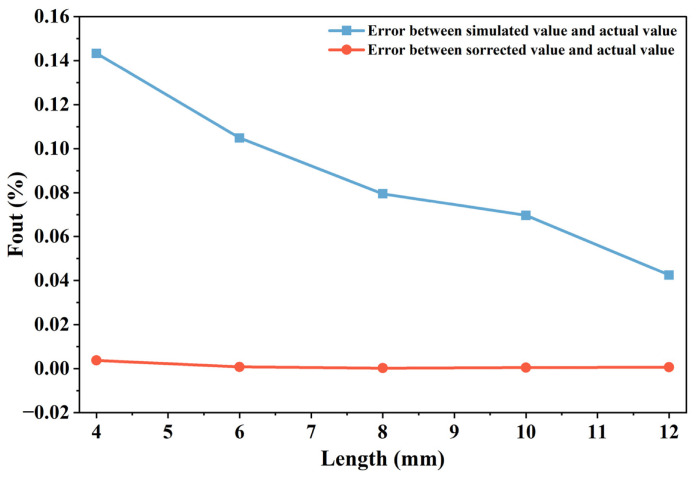
Comparison of defect length errors.

To assess the accuracy of the correction technique, adjustments are made to defect lengths measuring 4 mm, 6 mm, 8 mm, 10 mm, and 12 mm, derived from the theoretical calculations using the 3D magnetic dipole approach. These adjusted lengths are then evaluated against the true defect measurements, and the discrepancies between the adjusted and actual lengths are computed. The findings from this data comparison are presented in Table 4. Additionally, the discrepancies in the adjusted data are analyzed in relation to those of the unadjusted simulated defect lengths, with the results illustrated in Figure 37.

The data presented in Table 4 and Figure 37 show that the maximum deviation in identifying uncorrected simulated defect lengths can reach 0.58 mm, with all relative errors exceeding 4.00%. The average error is 8.80%, and the standard deviation of the errors is 3.38%. In contrast, the deviations for corrected lengths do not exceed 0.02 mm, and all relative errors remain below 0.30%, with an average error of 0.16% and a standard deviation of 0.05%, demonstrating a substantial improvement in identification accuracy and stability. Overall, the signal correction technique based on the midpoint of the $H^{\prime \prime }$ extreme values effectively mitigate peak offset interference caused by magnetic field diffusion and other factors, thereby significantly enhancing the accuracy of defect length identification and providing a more reliable method for quantitative defect assessment in MFL testing.

## 5. Experimental Research

**Figure 38:**
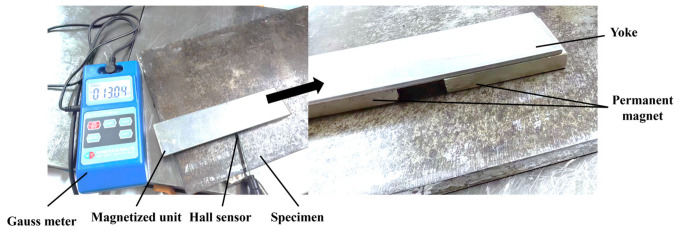
MFL testing platform.

**Figure 39:**
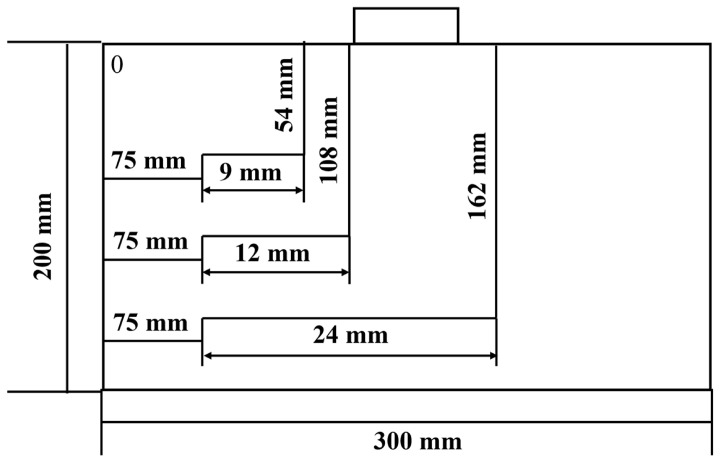
Geometric layout of the defects.

To ensure the accuracy of the simulation results, a testing platform for MFL has been developed, as illustrated in Figure 38. This platform is comprised of three essential elements: a gaussmeter for measuring magnetic field intensity, a magnetization unit for inducing magnetism in the specimen, and the specimen itself. The specimen is constructed from a 20# steel plate featuring surface cracks measuring 9 mm, 12 mm, and 24 mm in length. The arrangement of these defects is depicted in Figure 39.

### 5.1. Dynamic Scanning Detection Experiment

**Figure 40:**
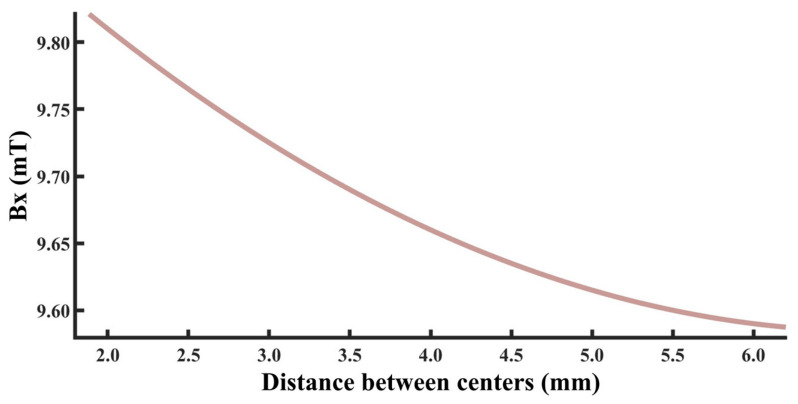
Fitting curve of dynamic scanning detection signals.

To assess the accuracy of the simulation results in dynamic scanning scenarios, a crack defect measuring 24 mm in length is examined with a fixed lift-off distance of 0.1 mm between the sensor and the specimen surface. The distances from the detector center to the defect center are established at 2 mm, 4 mm, and 6 mm. A gaussmeter is utilized to gauge the magnetic flux density, and the simulation findings are juxtaposed with the values obtained from experiments, allowing for calculation of discrepancies between the two. These findings are presented in Table 5. Furthermore, the experimental results are analyzed to develop a characterization model for dynamic scanning detection signals, which serves to validate the accuracy of the model derived from the simulation. The resulting fitted curve is illustrated in Figure 40.

Table 5 shows that, for various distances between detector and defect centers, the discrepancies between the simulated and measured values remain within 0.26 mm, with relative errors of less than 3.00%. The average error is 2.61%, and the standard deviation of the errors is 0.08%, confirming the high reliability of the simulation findings. Furthermore, Figure 40 shows that, as the relative distance between the defect and the Hall sensor increases, the MFL signal undergoes greater attenuation, resulting in a decrease in the magnetic flux density of the leakage field at the sensor location. The peak characterization model derived from fitting the dynamically scanned signal is $B_{x}=0.01x^{2}-0.14x+10.04$. This model conforms to the functional form identified in the simulation, thereby validating the accuracy of the simulation in characterizing the influence of distance between centers on the detection signal. Discrepancies in the specific coefficients may arise from factors such as ambient temperature and humidity, defect type, and the material properties of the specimen.

### 5.2. Detection Experiments with Different Lift-Off Values

**Figure 41:**
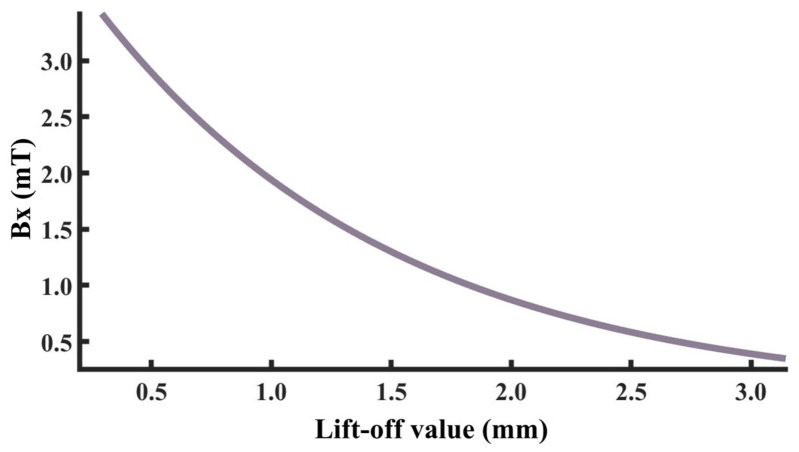
Fitting curve of detection signals with different lift-off values.

To assess the reliability of the simulation outcomes across various lift-off values, a 24 mm crack defect is selected for analysis. The lift-off values of the Hall sensor are configured at 0.1 mm, 0.5 mm, 1 mm, 1.5 mm, 2 mm, and 3 mm. The magnetic field strengths in defect-free and defect-present areas are measured separately, with the leakage magnetic field signal magnitude determined by their difference. The signal magnitudes derived from simulation and experiment are compared, and their relative discrepancies are calculated. These results are presented in Table 6. Furthermore, a characterization model of the sensor lift-off value for the detection signals is established by fitting the experimental data, with the fitted curve shown in Figure 41.

Table 6 shows that, for various lift-off distances, the discrepancies between the simulated and measured values remain below 0.12 mm, with all relative errors under 6%. The average error is 3.88%, and the standard deviation of the errors is 1.42%, indicating small errors and stable simulation results. Thus, the simulation results obtained from the established model across different lift-off distances are highly reliable. Furthermore, as shown in Figure 41, an increase in the lift-off distance enhances the diffusion of the magnetic field signal, leading to a decrease in the detection signal amplitude. The characterization model relating the lift-off value to the peak detection signal obtained by fitting is $B_{x}=4.32e^{-0.80x}-3869.50e^{-86.03x}$. This model conforms to the functional form derived from the simulation, confirming the reliability of the simulation in capturing the influence of lift-off distance on the detection signal. Discrepancies in the specific coefficients may be attributed to factors such as ambient temperature and humidity, defect type, and the material properties of the specimen.

### 5.3. Detection Experiments with Different Length Crack Defects

**Figure 42:**
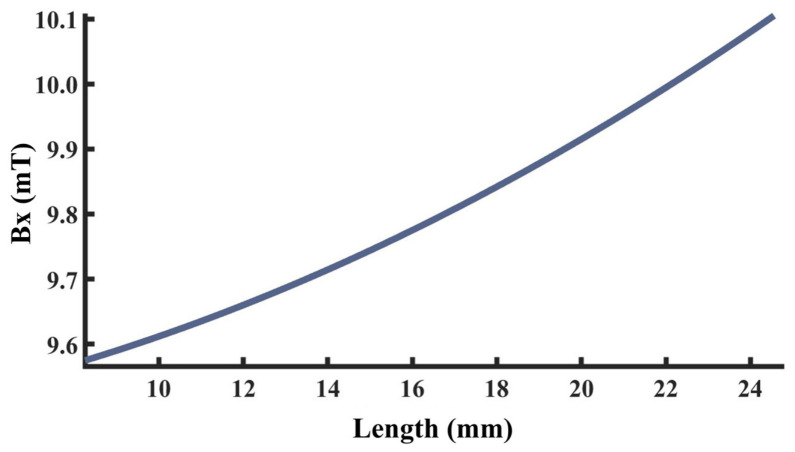
Fitting curve of detection signals at different crack lengths.

In order to assess the accuracy of the simulation outcomes for various defect lengths, specifically focusing on crack lengths of 9 mm, 12 mm, and 24 mm, a lift-off value of 0.1 mm is maintained. Both experimental measurements and simulations of magnetic field signals are conducted independently, followed by comparison of the two sets of values and computation of relative errors. The findings are presented in Table 7. Additionally, the experimental results are analyzed to create a model that characterizes crack length in relation to detection signals, with the resulting fitting curve illustrated in Figure 42.

Table 7 shows that, for the three defect lengths, the discrepancies between the simulated and measured values are all below 0.20 mm, with relative errors remaining under 2.00%. The average error is 1.84%, and the standard deviation of the errors is 0.13%, demonstrating that the established simulation model is capable of accurately and stably detecting defects of varying sizes, yielding highly reliable results. Furthermore, as shown in Figure 42, an increase in defect length reduces the distance between the defect edge and the permanent magnet, thereby strengthening the permanent magnetic field. Since the increase in the permanent magnetic field amplitude outweighs the reduction in the leakage magnetic field caused by enhanced diffusion and attenuation, the detection signal exhibits an upward trend as the defect length increases. The fitted characterization model relating crack length x to the peak detection signal $B_{x}$ is $B_{x}=0.64x^{3}-0.05x^{2}+1.00\times 10^{-3}x+6.80$. This model conforms to the functional form derived from the simulation, thereby confirming the accuracy of the simulation in characterizing the influence of defect length on the detection signal. Discrepancies in the specific coefficients may be attributed to factors such as ambient temperature and humidity, defect type, and the material properties of the specimen.

## 6. Conclusions

This study integrates systematic simulations, theoretical modeling, and experimental investigations to comprehensively examine MFL detection and quantitative defect assessment under supersaturated magnetization conditions. Through simulation comparison, the study determines the opposite magnetic pole configuration as the optimal magnetization scheme and clarifies the critical enhancing effect of supersaturated magnetization on MFL signals. When the magnetization intensity is increased to 3.0 T, the ferromagnetic material reaches an extremely high magnetic flux density state, and magnetic saturation at defect sites leads to more magnetic field lines “leaking” into the air, resulting in a significant increase in the leakage field signal amplitude compared to conventional saturated magnetization (1.85 T) and achieving better detection performance at the same lift-off value. This characteristic is particularly beneficial for the detection of micro-defects and deeply buried defects. In addition, the study quantifies the coupled influence laws of multiple parameters (sensor lift-off, defect burial depth, length, and depth) on the axial ($B_{x}$) and radial ($B_{z}$) MFL signals for rectangular defects, establishes a precise mathematical model, and reveals the physical essence of signal evolution from the perspective of magnetic charge distribution and spatial magnetic field diffusion. The effective detection thickness of the constructed detector is within 13 mm, and the detection capability of the detector for defects of different shapes is tested. To address the problem that traditional peak detection methods are susceptible to magnetic field diffusion interference, a rectangular defect length correction algorithm based on the midpoint of the extreme values of the second derivative of the axial signal is proposed. By extracting the abrupt change characteristics of signal curvature, this method effectively compensates for systematic errors, stably controlling the defect length identification error from over 10% with traditional methods to within 0.3%, significantly improving the accuracy and robustness of quantitative identification. Finally, theoretical verification using the 3D magnetic dipole model and multiple comparative experiments confirm the high reliability of the finite element simulation and the established characterization model (with an error margin of less than 6%), laying a solid foundation for the engineering application of supersaturated magnetization detection technology. The research materials in this paper are based on commonly used industrial carbon structural steels. Other ferromagnetic alloy materials will produce certain signal amplitude deviations due to differences in ferromagnetic performance parameters. However, for different materials, the formation mechanism of the leakage magnetic field caused by defects and the disturbance law of defects on the magnetic circuit are homologous. Therefore, after correcting the signal amplitude of the sensor parameters and detection signals in this paper, it can be extended to defect detection in other ferromagnetic materials.

In the future, the applicability and correction methods of the defect length correction algorithm based on the midpoint of the extreme values of the second derivative of the axial signal will be further investigated for non-typical double-peak characteristic defects such as irregular defects and applied to the correction of actual defect length detection. In addition, the application scope under complex working conditions will be expanded, and the defect signal characterization model will be further revised to enable the dimensional inversion of other defect shapes (e.g., ellipsoidal and irregular shapes) and the detection of defects in different ferromagnetic alloy materials. In addition, the influence mechanisms of material inhomogeneity and residual stress fields on detection signals will be further studied, and the design of supersaturated magnetizers will be carried out. At the same time, by leveraging the high-efficiency magnetization performance of supersaturated magnetization, the development of lightweight high-strength magnetization technologies suitable for high-speed dynamic detection, as well as the exploration of intelligent defect quantification and three-dimensional reconstruction methods based on multi-physical field information fusion and deep learning, will be the key to advancing this technology toward engineering and intelligent applications.

## Figures and Tables

**Table 1:** Statistical analysis of detection signal results.

	Indicator	Mean Value (mT)	Standard Deviation (mT)	Coefficient of Variation
Signal Type				
Axial Signal		9.36	1.79	19.12%
Radial Signal		9.92	1.54	15.52%

**Table 2:** Random perturbation experimental data.

Parameters	B (mT)	Parameters	B (mT)
d = 0.198 mm; l = 0.100 mm; μ = 300	16.47	d = 0.198 mm; l = 0.099 mm; μ = 300	16.48
d = 0.200 mm; l = 0.099 mm; μ = 300	16.57	d = 0.202 mm; l = 0.101 mm; μ = 300	16.21
d = 0.200 mm; l = 0.100 mm; μ = 297	16.70	d = 0.198 mm; l = 0.100 mm; μ = 297	16.61
d = 0.202 mm; l = 0.100 mm; μ = 300	16.23	d = 0.202 mm; l = 0.100 mm; μ = 303	16.09
d = 0.200 mm; l = 0.101 mm; μ = 300	16.55	d = 0.200 mm; l = 0.099 mm; μ = 297	16.71

**Table 3:** Comparison of theoretical and simulated data.

Actual Defect Length (mm)	Theoretical Value (mm)	Simulated Value (mm)	Error Between Simulated and Theoretical Values (%)
$2D_{x}=4$	3.46	3.43	0.87%
$2D_{x}=6$	5.53	5.37	2.89%
$2D_{x}=8$	7.43	7.36	0.94%
$2D_{x}=10$	9.43	9.30	1.38%
$2D_{x}=12$	11.53	11.49	0.35%

**Table 4:** Comparison of error data.

Actual Defect Length (mm)	Corrected Value (mm)	Error Between Simulated Value and Actual Value (%)	Error Between Corrected Value and Actual Value (%)
$2D_{x}=4$	3.99	14.25%	0.25%
$2D_{x}=6$	5.99	10.50%	0.17%
$2D_{x}=8$	8.01	8.00%	0.13%
$2D_{x}=10$	10.01	7.00%	0.10%
$2D_{x}=12$	12.02	4.25%	0.17%

**Table 5:** Comparison of simulation and measured data for the dynamic scanning experiments.

Distance Between Centers (mm)	Simulation Value (mT)	Measured Value (mT)	Relative Error (%)
2 mm	10.07	9.81	2.65%
4 mm	9.92	9.66	2.69%
6 mm	9.83	9.59	2.50%

**Table 6:** Comparison of simulation and measured data with different lift-off values.

Lift-Off Value (mm)	Simulation Value (mT)	Measured Value (mT)	Relative Error (%)
0.1	3.36	3.28	2.44%
0.5	3.00	2.91	3.09%
1.0	1.84	1.96	6.12%
1.5	1.16	1.23	5.38%
2.0	0.81	0.83	2.41%
3.0	0.50	0.52	3.85%

**Table 7:** Comparison of simulation and experimental data with crack defects of different lengths.

Length (mm)	Simulation Value (mT)	Measured Value (mT)	Relative Error (%)
9 mm	9.75	9.59	1.67%
12 mm	9.84	9.66	1.86%
24 mm	10.28	10.08	1.98%

## Data Availability

All data included in this study are available upon request via contacting the corresponding author.
